# Modeling cadmium-induced endothelial toxicity using human pluripotent stem cell-derived endothelial cells

**DOI:** 10.1038/s41598-017-13694-5

**Published:** 2017-11-01

**Authors:** Ling Tang, Jun Su, Ping Liang

**Affiliations:** 1 Key Laboratory of combined Multi-organ Transplantation, Ministry of Public Health, the First Affiliated Hospital, Zhejiang University, 310003 Hangzhou, China; 2 Institute of Translational Medicine, Zhejiang University, 310029 Hangzhou, China

## Abstract

Cadmium (Cd) is a harmful heavy metal that results in vascular diseases such as atherosclerosis. Prior evidence revealed that Cd induced endothelial cell (EC) death and dysfunction, supporting that ECs are a primary target of Cd-induced toxicity, and can cause severe pathologies of vascular diseases. However, the underlying mechanisms remain unclear. In this study, we investigated the mechanisms of Cd-induced endothelial toxicity in a human model system of H9 human pluripotent stem cell-derived endothelial cells (H9-ECs). We showed that H9-ECs were susceptible to CdCl_2_ induction, leading to detrimental changes of cell structure and significantly elevated level of apoptosis. We demonstrated that CdCl_2_-treated H9-ECs gave rise to a clear EC dysfunction phenotype and significantly differential transcriptomic profile. Signaling pathway analysis revealed that P38 or ERK signaling pathway is critical to cadmium-induced EC apoptosis and dysfunction, and inhibition of P38 or ERK effectively rescued CdCl_2_-induced endothelial toxicity in H9-ECs. Conclusively, hPSC-ECs can be a reliable model to recapitulate the EC pathological features and transcriptomic profile, which may provide a unique platform for understanding the cellular and molecular mechanisms of Cd-induced endothelial toxicity and for identifying therapeutic drugs for Cd-induced vascular diseases.

## Introduction

Cadmium (Cd) is a soft, malleable, ductile and bluish-white divalent metal, which is widely used by electric batteries, pigments, coatings and electroplating^1–5^. Cd is thought to be a serious environmental toxicant and harmful to the health of humans, which is specifically listed in the European Restriction of Hazardous Substances^6^. The British Geological Survey reports that in 2001, China was the top producer of cadmium with almost one-sixth of the world’s production. The primary target organs of Cd include kidney, liver, bone, intestine, brain and cardiovascular systems^7–12^.

Cd-induced toxicity has been widely studied and Cd can induce apoptosis in various cell types^13–16^. Growing evidence suggests that elevated serum levels of Cd correlate with risk of vascular diseases and endothelial cells (EC) are one of the primary targets of Cd-induced cytotoxicity, leading to vascular diseases such as atherosclerosis^17,18^. However, the molecular mechanisms of Cd-induced endothelial toxicity have not been well studied yet.

In recent years, human pluripotent stem cells (hPSCs) have been thought as a potentially ideal cell resource for translational and regenerative medicine^19–22^. Differentiation of hPSCs into functional ECs (hPSC-ECs) provides easy-accessible, unlimited, reproducible and physiologically relevant source of cells for vascular disease modeling, drug testing and transplantation therapy^23–25^.

In this study, we first investigated if hPSC-ECs can serve as a model to recapitulate the Cd-induced endothelial toxicity *in vitro*. We then demonstrated a clear EC dysfunction phenotype and significantly differential transcriptomic profile. Further studies revealed that P38 or ERK signaling pathways is critical to Cd-induced EC apoptosis as well as EC dysfunction, and inhibition of P38 or ERK effectively rescued the Cd-induced endothelial toxicity in hPSC-ECs. Therefore, successful establishment of such a cellular model may provide a unique platform for understanding the cellular and molecular mechanisms of Cd-induced endothelial toxicity, and for identifying drugs for Cd-induced vascular diseases.

## RESULTS

### Generation and characterization of endothelial cells derived from H9 embryonic stem cells

**Figure 1:**
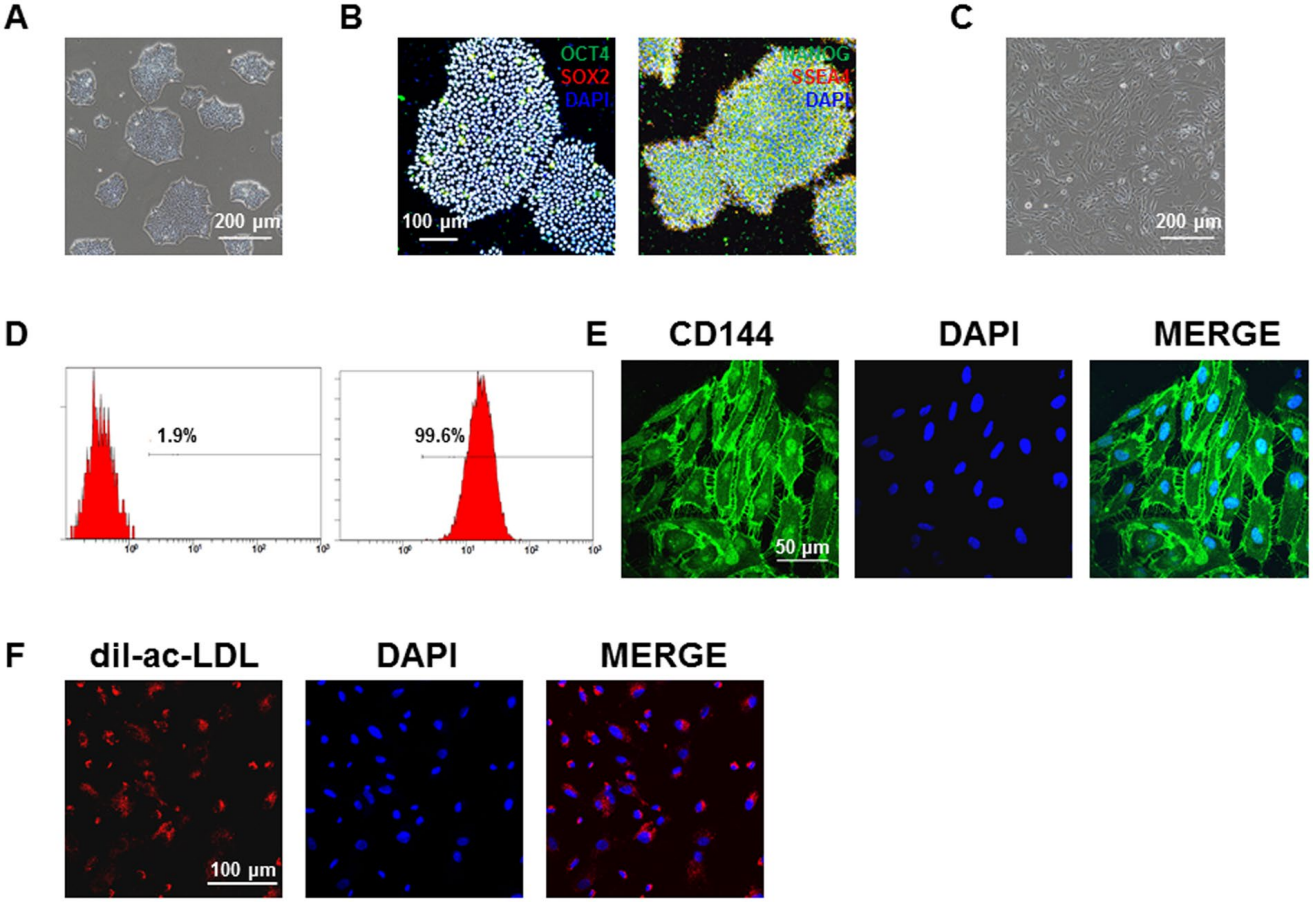
Generation and characterization of endothelial cells derived from H9 human embryonic stem cells. (**A**) Typical morphology of undifferentiated H9 hESCs. Scale bar, 200 μm. (**B**) Pluripotent staining of H9 hESCs using OCT4 (Green), SOX2 (Red), NANOG (Green) and SSEA4 (Red). DAPI indicates nuclear staining (Blue). Scale bar, 100 μm. (**C**) Typical morphology of H9-ECs. Scale bar, 200 μm. (**D**) FACS analysis of CD144-positive cells. (**E**) CD144 (Green) staining of H9-ECs. DAPI indicates nuclear staining (Blue). Scale bar, 50 μm. (**F**) Dil-ac-LDL (Red) staining of H9-ECs. DAPI indicates nuclear staining (Blue). Scale bar, 100 μm.

H9 embryonic stem cells (H9) were selected for generation of endothelial cells (ECs), which exhibited stem cell morphology and expressed pluripotency markers such as OCT4, NANOG, SOX2 and SSEA-4 (Fig. 1A,B and Supplemental Fig. 1). Using an *in vitro* monolayer endothelial differentiation protocol, we successfully differentiated H9 into ECs. On day 10 of induction of differentiation, we observed dramatically morphological change towards to ECs (Fig. 1C). CD144 positive cells were subsequently sorted by MACS, which gave rise to a purification of 99.6% (Fig. 1D). The sorted cells were then plated on 0.1% matrigel-coated plates for downstream expansion and characterization. The isolated H9-ECs showed positive staining of endothelial-specific marker CD144, as well as dil-ac-LDL uptake (Fig. 1E,F).

### Cadmium induces cell damage and apoptosis in H9-ECs

**Figure 2:**
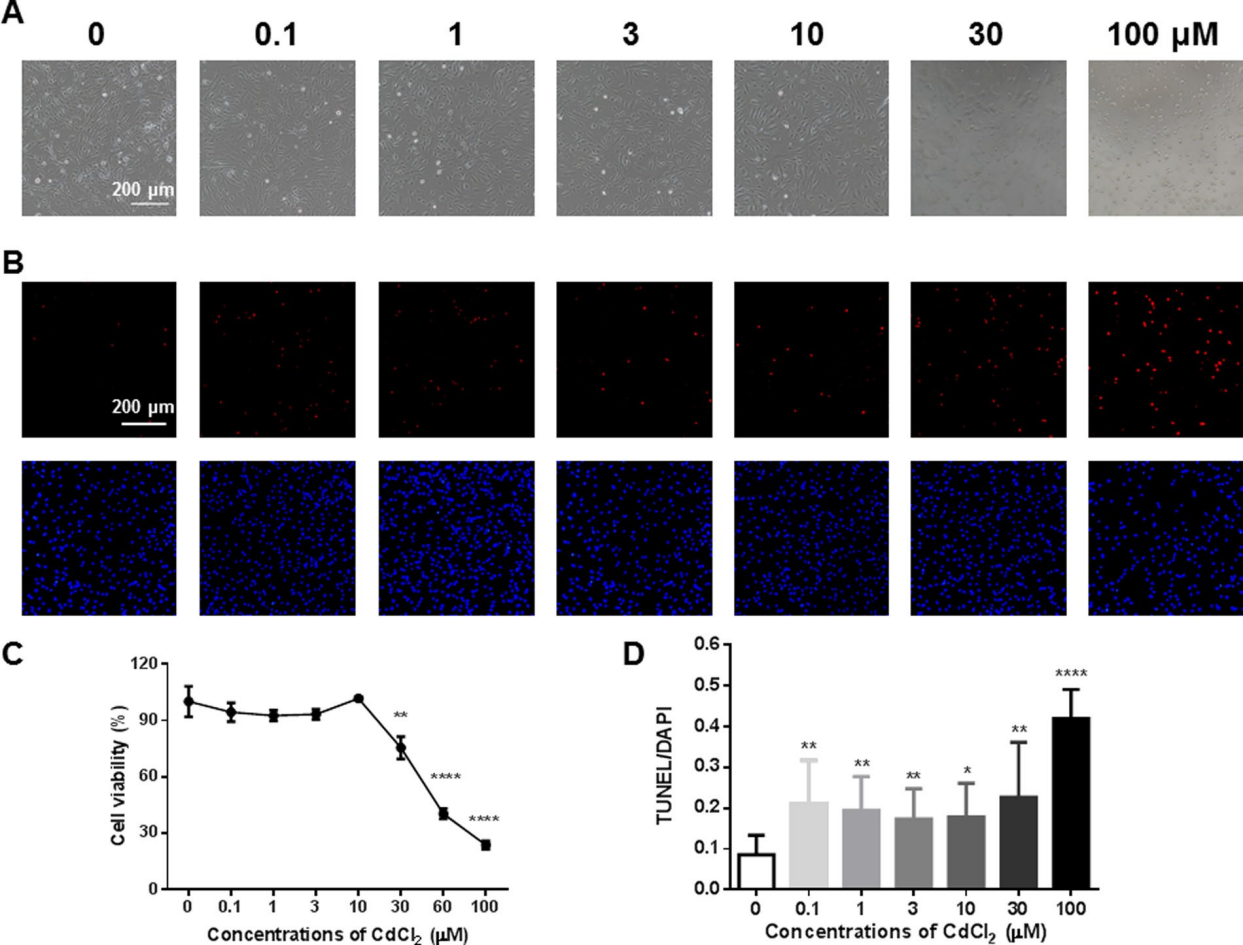
Cadmium induces cell damage and apoptosis in H9-ECs. (**A**) Representative images of morphological changes in H9-ECs induced by escalating doses of CdCl_2_ for 24 h. Scale bar, 200 μm. (**B**) Representative confocal images of TUNEL and DAPI staining in control and CdCl_2_-treated H9-ECs. Scale bar, 200 μm. (**C**) Comparison of cell viability between control and CdCl_2_-treated H9-ECs. ^**^ *P* < 0.01 and ^****^ *P* < 0.0001. (**D**) Bar graph to compare the ratio of TUNEL/DAPI between control and CdCl_2_-treated cells. ^*^ *P* < 0.05, ^**^ *P* < 0.01 and ^****^ *P* < 0.0001.

**Figure 3:**
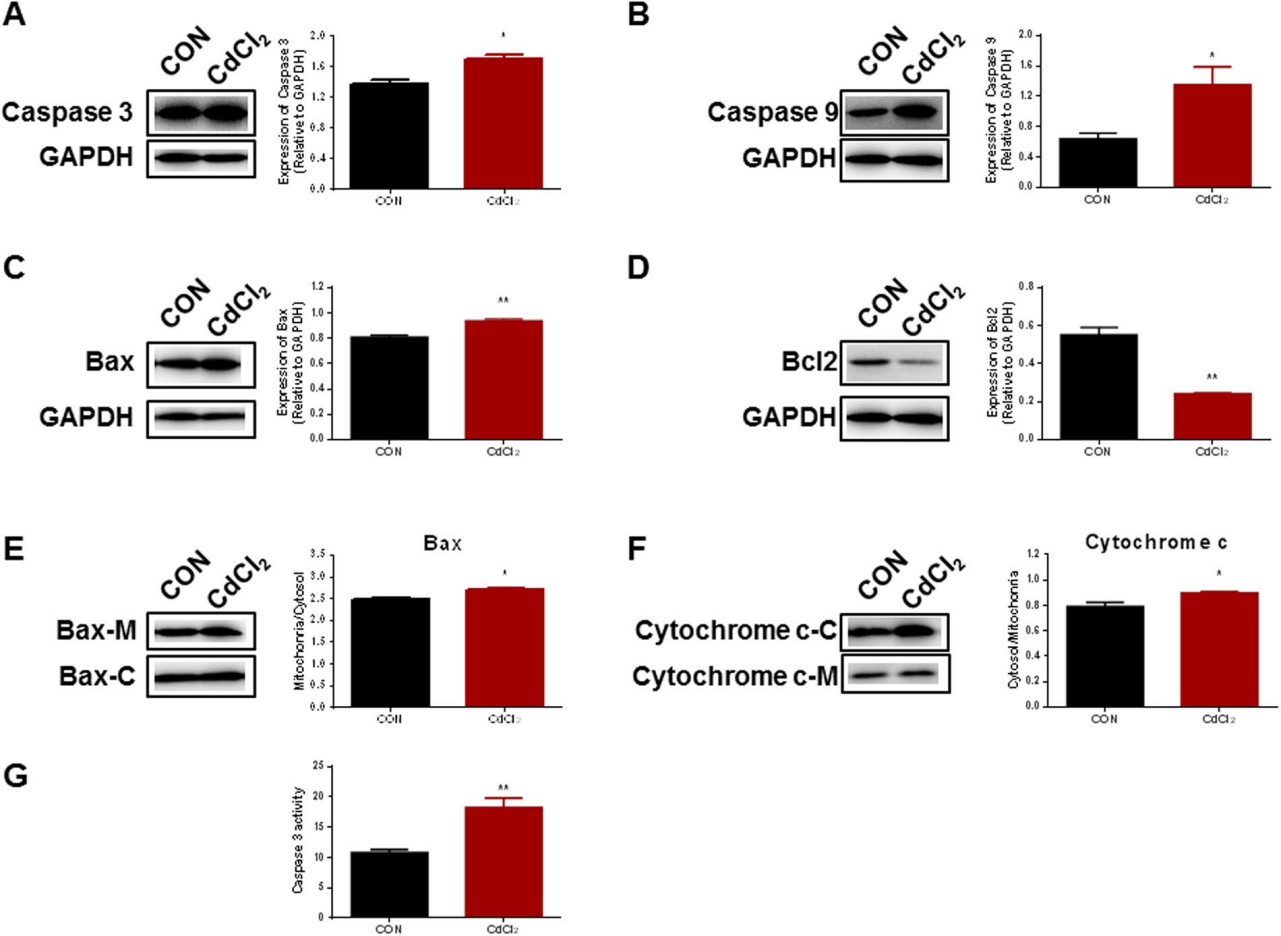
Confirming cadmium-induced apoptosis in H9-ECs. (**A**–**D**). Left panel, western blot analysis of Caspase 3, Caspase 9, Bax and Bcl2 expression in control and CdCl_2_-treated H9-ECs. GAPDH is used for the loading control. Full-length blots are shown in Supplemental Figs 4–7; Right panel, bar graph to compare the expression of Caspase 3, Caspase 9, Bax and Bcl2 between control and CdCl_2_-treated cells. ^*^ *P* < 0.05, ^**^ *P* < 0.01. (**E**). Left panel, western blot analysis of Bax-Mitochondria/Bax-cytosol in control and CdCl_2_-treated H9-ECs. Full-length blots are shown in Supplemental Fig. 8; Right panel, bar graph to compare Bax-Mitochondria/Bax-cytosol between control and CdCl_2_-treated cells. ^*^ *P* < 0.05. Bax-M and Bax-C denote Bax-Mitochondria and Bax-Cytosol, respectively. (**F**). Left panel, western blot analysis of Cytochrome c-cytosol/Cytochrome c-Mitochondria in control and CdCl_2_-treated H9-ECs. Full-length blots are shown in Supplemental Fig. 9; Right panel, bar graph to compare Cytochrome c-cytosol/Cytochrome c-Mitochondria between control and CdCl_2_-treated cells. ^*^ *P* < 0.05. Cytochrome c-M and Cytochrome c-C denote Cytochrome c-Mitochondria and Cytochrome c-Cytosol, respectively. (**G**) Comparison of Caspase 3 activity between control and CdCl_2_-treated H9-ECs. ^**^ *P* < 0.01.

H9-ECs were exposed to escalating dosages of cadmium chloride (CdCl_2_) from 0.1 μM to 100 μM for 24 h, and we observed dramatic morphological changes and cell damage in H9-ECs at high doses of CdCl_2_ treatment (30 and 100 μM) (Fig. 2A and Supplemental Fig. 2). We observed a significantly reduced cell viability in H9-ECs started from 30 μM CdCl_2_ treatment, when compared to control cells (Fig. 2C). We next performed TUNEL assay to investigate if the CdCl_2_-induced morphological changes and cell damage were associated with apoptosis. We observed a significantly increased ratio of TUNEL-positive cells in CdCl_2_-treated H9-ECs started from 0.1 μM, as compared to control cells (Fig. 2B,D and Supplemental Fig. 3). In line with the TUNEL data, the expression of Caspase 3, Caspase 9 and Bax were all significantly increased whereas the expression of Bcl2 was significantly reduced in 30 μM CdCl_2_-treated H9-ECs, when compared to controls (Fig. 3A–D and Supplemental Figs 4–7). Interestingly, we observed translocation of Bax from cytosol to mitochondria as well as translocation of Cytochrome c from mitochondria to cytosol in H9-ECs treated with 30 μM CdCl_2_ (Fig. 3E,F and Supplemental Figs 8,9). Moreover, we observed significantly increased Caspase 3 activity in 30 μM CdCl_2_-treated H9-ECs (Fig. 3G). H9-ECs were further stained by Propidium Iodide (PI) and flow cytometry analysis demonstrated increased fraction of sub-G1 in 30 μM CdCl_2_-treated cells (Supplemental Figure 10). We chose 30 μM CdCl_2_ with significantly reduced cell viability and strong TUNEL signal as the induction dosage for the downstream investigations. Taken together, these data suggest that H9-ECs are susceptible to CdCl_2_ induction, leading to detrimental changes of cell structure, reduced cell viability and increased apoptosis.

### Cadmium leads to endothelial dysfunction in H9-ECs

**Figure 4:**
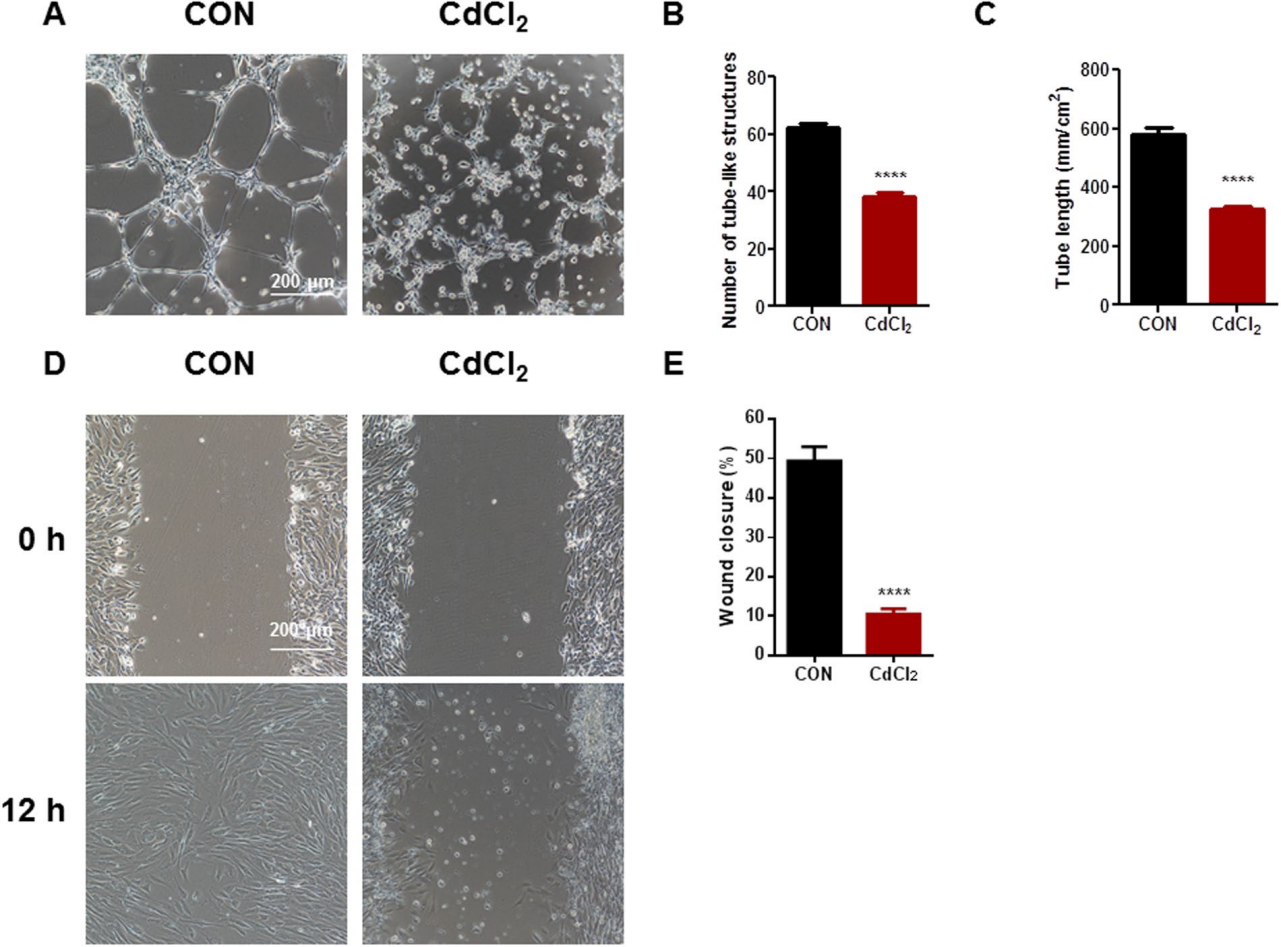
Cadmium leads to endothelial dysfunction in H9-ECs. (**A**) Representative images of tube formation on matrigel in control and CdCl_2_-treated H9-ECs assessed at 6 h. (**B**) Bar graph to compare the number of tube-like structures between control and CdCl_2_-treated cells. ^****^ *P* < 0.0001. (**C**) Bar graph to compare tube length between control and CdCl_2_-treated cells. ^****^ *P* < 0.0001. (**D**) Representative images of wound closure in control and CdCl_2_-treated H9-ECs assessed at 12 h. (**E**) Bar graph to compare the percentage of wound closure between control and CdCl_2_-treated cells. ^****^ *P* < 0.0001.

Having establishing the cell model of cadmium-induced endothelial toxicity, we next determined if the function of H9-ECs altered with CdCl_2_ induction. The CdCl_2_-treated H9-ECs showed significantly decreased tube formation capacity compared to control cells after 6 h, with reduced numbers of tube-like structures (Control: 62.00 ± 1.83; CdCl_2_: 38.00 ± 1.58) and reduced tube length (Control: 579.00 mm/cm^2^ ± 12.66; CdCl_2_: 324.12 mm/cm^2^ ± 7.20) (Fig. 4A–C and Supplemental Figure 11). Moreover, the CdCl_2_-treated H9-ECs showed decreased migration detected by a wound closure scratch assay when compared to control cells (Control: 49.32% ± 1.79; CdCl_2_: 10.53 ± 0.64) (Fig. 4D,E and Supplemental Figure 12). There is no significant difference of cell proliferation between control and CdCl_2_-treated cells (Supplemental Figure 13). Collectively, these results demonstrate that the H9-ECs exhibit a distinct endothelial dysfunction phenotype in response to CdCl_2_ treatment.

### RNA-Seq analysis of CdCl2-treated H9-ECs

**Figure 5:**
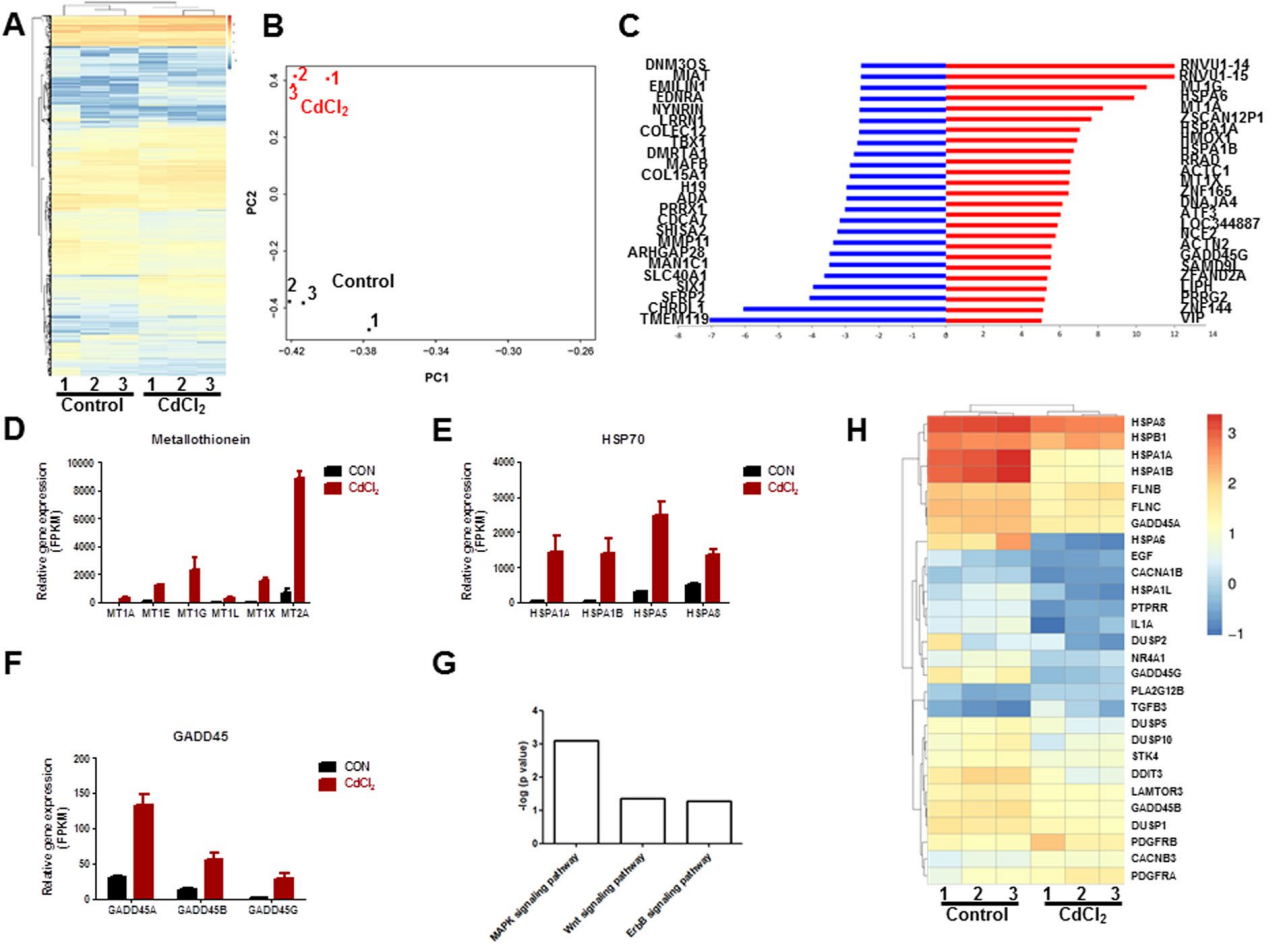
RNA-Seq analysis of CdCl_2_-treated H9-ECs. (**A**) Heatmap demonstrating the differential gene expression pattern between control and CdCl_2_-treated H9-ECs. (**B**) Principal component analysis (PCA) revealed that CdCl_2_-treated samples clustered separately from control ones. (**C**) Top 20 genes showing the greatest differences in expression between CdCl_2_-treated and control H9-ECs. Up-regulated genes are shown in red and down-regulated genes are shown in blue. (**C–E**) Bar graphs to compare the FPKM values of metallothionein (MT), HSP70, and GADD45 family genes between control and CdCl_2_-treated H9-ECs. (**F**) Ingenuity pathway analysis (IPA) showing significantly altered signaling pathways in CdCl_2_-treated H9-ECs compared to control cells. (**G**) Heatmap demonstrating the differential gene expression associated with MAPK signaling pathway between control and CdCl_2_-treated H9-ECs.

To further uncover the molecular mechanisms of cadmium-induced endothelial toxicity in a human-based environment, we next performed genome-wide RNA sequencing (RNA-Seq) by comparing paired control and CdCl_2_-treated H9-ECs from 3 independent endothelial differentiations (Fig. 5A). Principal component analysis (PCA) revealed that CdCl_2_-treated samples clustered separately from control ones (Fig. 5B). We observed that 1145 genes out of 19676 total genes (722 up-regulated, and 423 down-regulated) were differentially expressed in CdCl_2_-treated H9-ECs when compared to control cells (Fig. 5C). Among these, metallothionein isoforms including MT1A, MT1E, MT1G, MT1L, MT1X and MT2A were dramatically up-regulated, which were associated with cadmium-induced cytotoxicity (Fig. 5D)^26^. Noticeably, a cluster of heat shock protein member A (HSP70) gene family members including HSPA1A (also named HSP70A), HSPA1B (also named HSP70B), HSPA5 and HSPA8 was amongst the genes up-regulated, which were associated with endothelial function (Fig. 5E)^27,28^. Moreover, we identified growth arrest and DNA-damage-inducible protein (GADD45) genes including GADD45A, GADD45B and GADD45G were also significantly up-regulated, which have been implicated in stress signaling to activate several stress response kinase such as P38 MAPK, thus resulting in apoptosis (Fig. 5F)^29^. Interestingly, gene ontology (GO) analysis revealed that genes were positively enriched in “blood vessel development”, “blood vessel morphogenesis”, “vasculature development”, “angiogenesis”, and “regulation of endothelial cell migration”, which are highly consistent with observed CdCl_2_-induced EC dysfunction (Supplemental Figure 14). Ingenuity pathway analysis (IPA) demonstrated significant up-regulation of mitogen-activated kinase (MAPK), as well as Wnt and ErbB signaling pathways (Fig. 5G–H).

### P38 and ERK1/2 MAPK signaling pathways are critical to cadmium-induced cell apoptosis and endothelial dysfunction in H9-ECs

**Figure 6:**
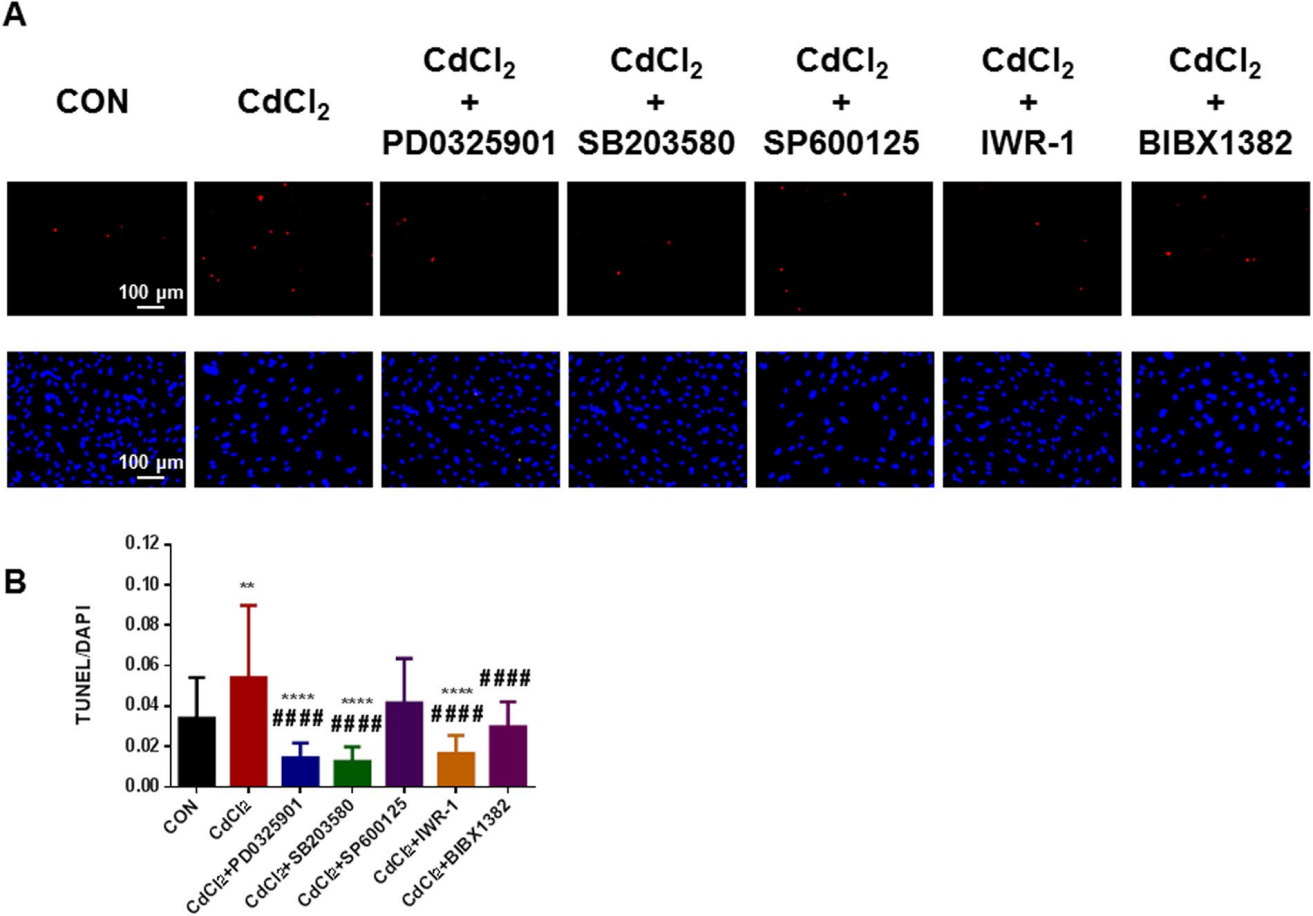
Rescuing CdCl_2_-induced cell apoptosis in H9-ECs using a panel of inhibitors. (**A**) Representative confocal images showing the rescuing effect of CdCl_2_-induced apoptosis in H9-ECs by a panel of inhibitors to block ERK, P38, JNK, Wnt and ErbB, respectively. (**B**) Bar graph to compare the ratio of TUNEL/DAPI between different groups in (**A**). **P < 0.01, ****P < 0.0001, when compared to control cells; ^####^ *P* < 0.0001, when compared to CdCl_2_-treated cells.

**Figure 7:**
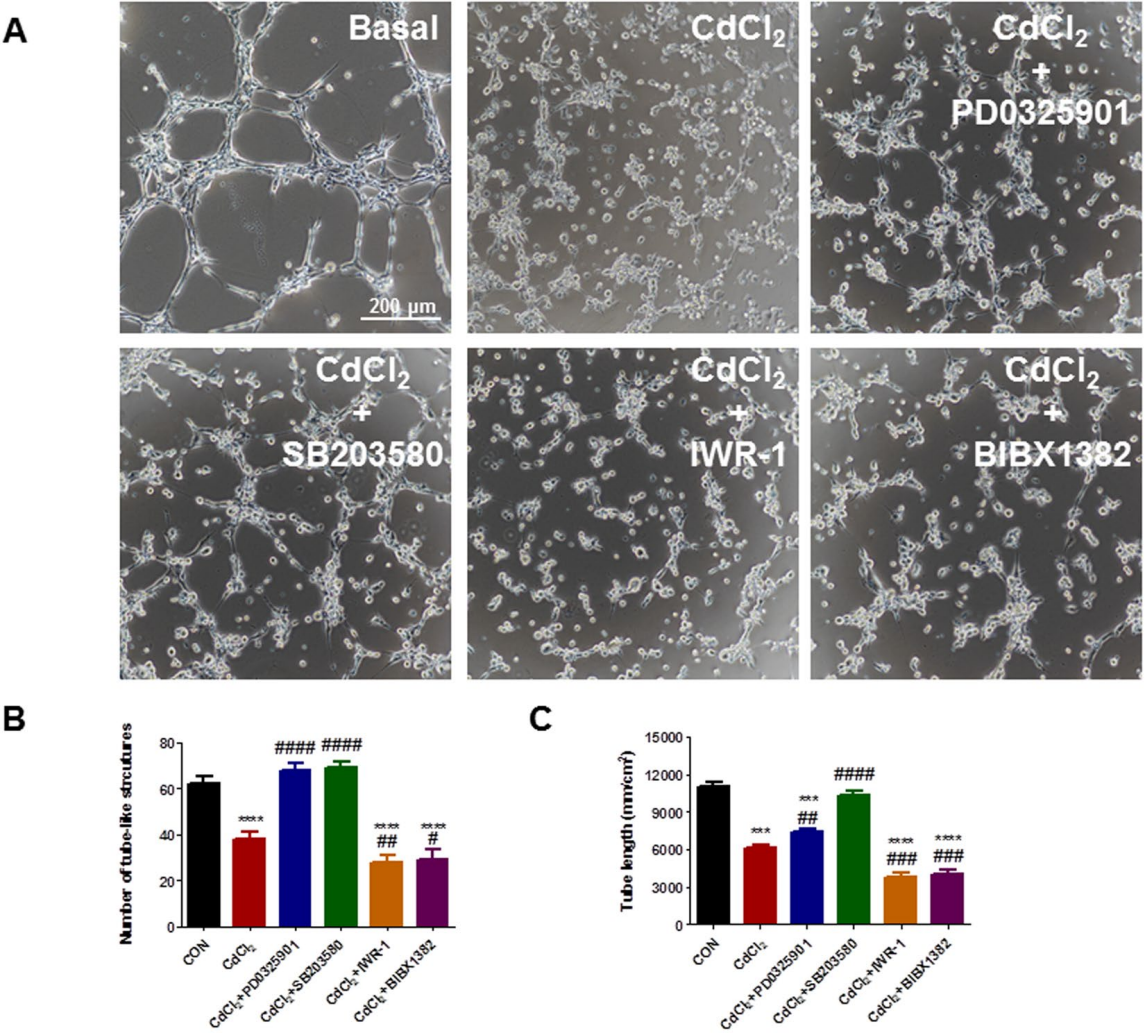
P38 and ERK signaling pathways are critical to cadmium-induced tube formation dysfunction in H9-ECs. (**A**) Representative confocal images showing the rescuing effect of CdCl_2_-induced tube formation dysfunction phenotype in H9-ECs by a panel of inhibitors to block ERK, P38, Wnt and ErbB, respectively. (**B–C**) Bar graphs to compare the number of tube-like structures and tube length between different groups in (**A**). ^***^ *P* < 0.001, ^****^ *P* < 0.0001, when compared to control cells; ^#^ *P* < 0.05, ^##^ *P* < 0.01, ^###^ *P* < 0.001, ^####^ *P* < 0.0001, when compared to CdCl_2_-treated cells.

**Figure 8:**
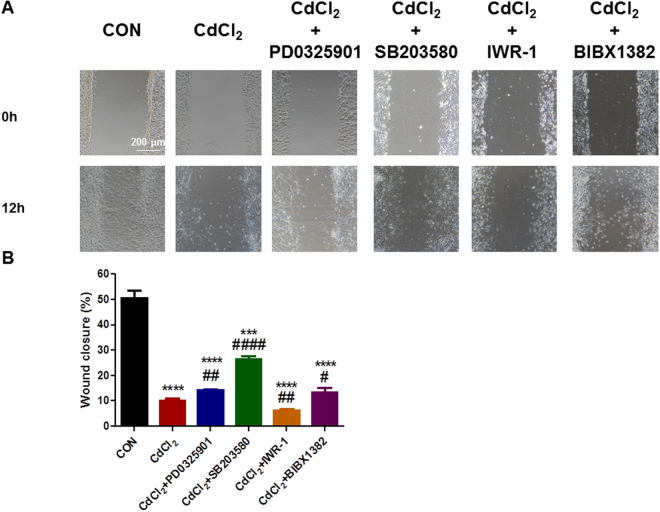
P38 and ERK signaling pathways are critical to cadmium-induced migration dysfunction in H9-ECs. (**A**) Representative confocal images showing the rescuing effect of CdCl_2_-induced migration dysfunction phenotype in H9-ECs by a panel of inhibitors to block ERK, P38, Wnt and ErbB, respectively. (**B**) Bar graph to compare the percentage of wound closure between different groups in (**A**). ^***^ *P* < 0.001, ^****^ *P* < 0.0001, when compared to control cells; ^#^ *P* < 0.05, ^##^ *P* < 0.01, ^####^ *P* < 0.0001, when compared to CdCl_2_-treated cells.

**Figure 9:**
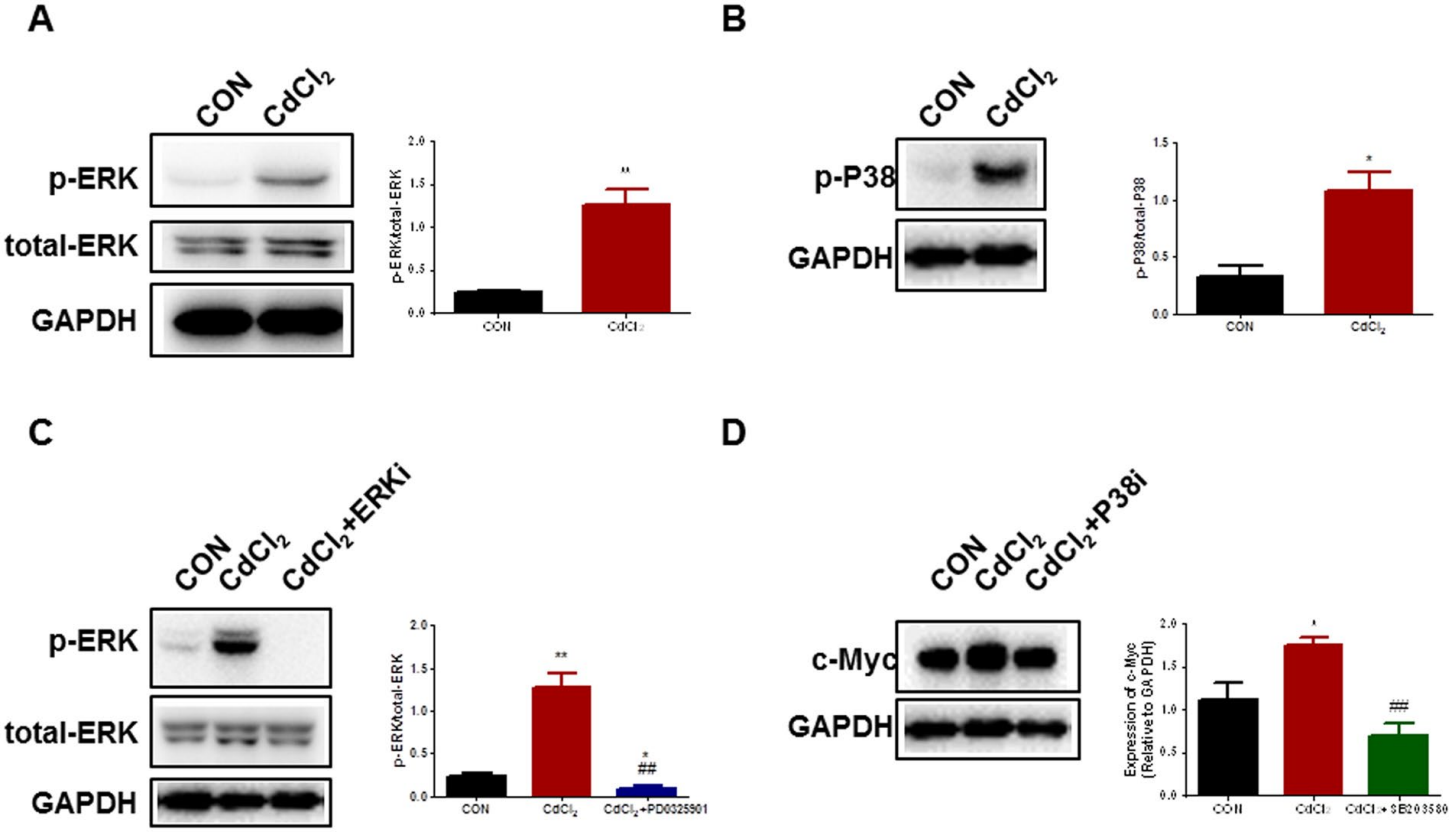
Suppression of elevated protein expression of P38 or ERK1/2 in H9-ECs induced by CdCl_2_ by targeting P38 or ERK signaling pathway. (**A**) Left panel, western blot analysis of p-ERK expression in control and CdCl_2_-treated H9-ECs. GAPDH is used for the loading control. Full-length blots are shown in Supplemental Figure 18; Right panel, bar graph to compare the p-ERK expression between control and CdCl_2_-treated cells. ^**^ *P* < 0.01. (**B**) Left panel, western blot analysis of p-P38 expression in control and CdCl_2_-treated H9-ECs. GAPDH is used for the loading control. Full-length blots are shown in Supplemental Figure 19; Right panel, bar graph to compare the p-P38 expression between control and CdCl_2_-treated cells. ^*^ *P* < 0.05. (**C**) Left panel, representative gel image showing PD0325901 (ERKi, ERK signaling pathway inhibitor) significantly decreased the CdCl_2_-induced elevated expression of p-ERK in H9-ECs. GAPDH is used for the loading control. Full-length blots are shown in Supplemental Figure 20; Right panel, bar graph to compare the p-ERK expression between different groups. ^*^ *P* < 0.05, ^**^ *P* < 0.01, when compared to control cells; ^##^ *P* < 0.01, when compared to CdCl_2_-treated cells. (**D**) Left panel, representative gel image showing SB203580 (P38i, P38 signaling pathway inhibitor) significantly decreased the CdCl_2_-induced elevated expression of c-Myc in H9-ECs. GAPDH is used for the loading control. Full-length blots are shown in Supplemental Figure 21; Right panel, bar graph to compare the c-Myc expression between different groups. ^*^ *P* < 0.05, when compared to control cells; ^##^ *P* < 0.01, when compared to CdCl_2_-treated cells.

To test whether abnormal activation of MAPK (Extracellular signal–regulated kinases (ERK), P38 and c-Jun N-terminal kinases (JNK)), Wnt, or ErbB signaling pathway gave rise to functional consequences, we therefore examined if inhibition of specific signaling pathways can rescue CdCl_2_-induced apoptosis and endothelial dysfunction in H9-ECs. TUNEL assays were performed to detect cell apoptosis and we found that ERK inhibitor PD0325901, P38 inhibitor SB203580, Wnt inhibitor IWR-1, or ErbB inhibitor BIBX1382 significantly reduced the CdCl_2_-induced apoptosis, whereas JNK inhibitor SP600125 did not (Fig. 6A,B and Supplemental Figure 15). Furthermore, the endothelial dysfunction phenotype of CdCl_2_-treated H9-ECs was rescued by inhibition of P38 or ERK but not Wnt or ErbB, with restored tube formation (Fig. 7A–C and Supplemental Figure 16) and migration capacities (Fig. 8A,B and Supplemental Figure 17) similar to control cells. These data were in line with previous studies in which MAPK signaling pathway may be involved in cadmium-induced toxicity in mouse brain microvascular endothelial cells^30,31^. To confirm the enhanced activation of P38 and ERK signaling pathways, we next performed western blot to compare the protein expression levels of phosphorylated P38 (p-P38) and phosphorylated ERK1/2 (p-ERK1/2). Consistently, CdCl_2_-treated H9-ECs showed significantly higher levels of p-P38 and p-ERK1/2 compared to control cells, indicating activation of p38 and ERK signaling pathways in response to cadmium (Fig. 9A,B and Supplemental Figure 18,19). However, when treated with SB203580, a selective P38 signaling pathway inhibitor by targeting MAPKAPK2 and MAPKAPK3, the cells demonstrated significantly reduced expression level of c-Myc, which is downstream target of MAPKAPK2/MAPKAPK3 (Fig. 9C and Supplemental Figure 20). Similarly, PD0325901 which specifically inhibits ERK signaling pathway inhibitor by targeting ERK1/2, resulted in significantly decreased expression level of p-ERK1/2 (Fig. 9D and Supplemental Figure 21). Taken together, our results indicate that P38 or ERK signaling pathway is involved and critical to cadmium-induced EC apoptosis and dysfunction, and inhibition of P38 and ERK effectively rescued CdCl_2_-induced endothelial toxicity in H9-ECs.

## Discussion

Cadmium (Cd), a harmful heavy metal, results in vascular diseases such as atherosclerosis. Prior studies have revealed that Cd induced endothelial cell (EC) death and dysfunction, supporting that EC is a primary target of Cd-induced toxicity to cause severe pathologies of vascular diseases^17,18^. However, the molecular mechanisms of Cd-induced EC toxicity remain unclear.

In this study, we used a human ESC-EC model to investigate the molecular mechanisms underlying the EC apoptosis and dysfunction induced by cadmium exposure. We observed CdCl_2_-induced apoptosis at low dose of 0.1 μM, suggesting that H9-EC model is susceptible to cadmium induction and could a suitable model to study cadmium-induced EC toxicity. Our results showed that CdCl_2_-treated H9-ECs were susceptible to CdCl_2_ induction and displayed detrimental changes of cell structure and significantly elevated level of apoptosis.

We also identified cadmium-induced EC dysfunction in H9-ECs. CdCl_2_-treated H9-ECs demonstrated attenuated capacities of tube formation and migration, whereas proliferation was comparable between control and CdCl_2_-treated H9-ECs. Our results provided a comprehensive investigation of the CdCl_2_-induced endothelial phenotype, which recapitulated human endothelial physiology and enhanced our understanding of the mechanism of CdCl_2_-induced endothelial toxicity.

An observation has not been previously reported was the transcriptomic feature in H9-ECs exposed to Cd. We demonstrated a significantly differential gene expression profile in H9-ECs exposed to CdCl_2_ when compared to control cells, in which 722 genes were up-regulated and 423 were down-regulated. Among these, several isoforms of metal binding protein metallothionein (MT1A, MT1E, MT1G, MT1L, MT1X and MT2A) and a cluster of HSP70 gene family members (HSPA1A, HSPA1B, HSPA5 and HSPA8) showed an increase in gene expression, which may serve as cellular defensive mechanisms of ECs in response to cadmium^32^. Previous studies have investigated the signaling pathways associated with cadmium-induced apoptosis in different cell types including ECs. Zhang *et al*. reported that NF-κB pathway plays an essential role in maintaining the survival of Cd-exposed human renal glomerular endothelial cells^33^. Nazimabashir *et al*. reported that grape seed proanthocyanidins attenuates cadmium-induced membrane disturbances, apoptosis and oxidative stress in rat cardiomyocytes through Nrf2 signaling pathway^34^. Jiang *et al*. reported that MAPK and PI3K/Akt signaling pathways are associated with cadmium-induced astrocyte cytotoxicity and cell death in mice^35^. Xu *et al*. reported that MAPK and mTOR signaling pathways are associated with cadmium-induced neuronal apoptosis in mice^36^. Chen *et al*. reported that CaMK II is involved in cadmium-induced activation of MAPK and mTOR pathways leading to neuronal cell death^37^. Son *et al*. reported that cadmium induces intracellular Ca^2+^ and H_2_O_2_-dependent apoptosis through JNK- and P53-mediated pathways in mouse skin epidermal cell line^38^. Zou *et al*. reported that salidroside protects against cadmium-induced hepatotoxicity in rats via GJIC and MAPK pathways^39^. Importantly, we demonstrated that MAPK, Wnt and ErbB signaling pathways were markedly altered. Moreover, using specific blocker to inhibit certain pathway, we found that inhibition of ERK, P38, Wnt, or ErbB effectively protected the CdCl_2_-induced apoptosis in H9-ECs detected by TUNEL assay. This is in line with previous evidence that P38 signaling pathway is activated in cadmium-induced cell apoptosis. Interestingly, P38 or ERK but not Wnt or ErbB inhibitor effectively rescued EC dysfunction phenotype, with restored tube formation as well as migration capacities. Overall, our results highlighted the ability of H9-ECs to provide insight into the abnormal EC function phenotype in response to cadmium, which may be mediated through P38 and ERK signaling pathways.

In summary, our data provided insight into cellular mechanisms of cadmium-induced endothelial cell toxicity. We identified CdCl_2_-treated H9-EC phenotypic features including elevated level of apoptosis, attenuated capacity of tube formation, attenuated capacity of migration, and differential transcriptomic profile. We highlighted P38 and ERK signaling pathway was markedly elevated in CdCl_2_-treated H9-ECs and inhibition of P38 and ERK effectively rescued the EC apoptosis and dysfunction induced by cadmium. Our model provided improved knowledge of cellular phenotype associated with Cd-induced EC toxicity, allowing us to enhance the understanding of Cd-induced vascular disease mechanisms and screen therapeutic drug targets.

## Methods

### Culture and maintenance of H9

H9 human embryonic stem cells (hESCs) were obtained from WiCELL (Madison, WI) and were chosen for use in this study. H9 hESCs were maintained in feeder-free mTeSR1 (STEMCELL) media on matrigel-coated (BD Biosciences) plates at 37 °C with 5% (vol/vol) CO_2_. The media were daily changed and cells were passaged every 3–4 days using accutase (StemPro).

### Differentiation of H9-derived endothelial cells

H9 hESCs were differentiated into endothelial cells (ECs) using a 2D monolayer differentiation protocol as previously described^40^. On day 0, H9 were placed in differentiation medium (RPMI and B-27 supplement minus insulin) (GIBCO) with 6 μM CHIR-99021 (Axon Medchem) for 2 days, followed by differentiation medium with 3 μM CHIR-99021 for another 2 days. On Day 4, the medium was changed to differentiation medium with 50 ng/ml vascular endothelial growth factor (VEGF; PeproTech) and 10 ng/ml fibroblast growth factor basic (FGF-b; PeproTech) for 5 days. On Day 9, H9-ECs were sorted for CD144^+^ by MACS and cultured in EGM-2 medium (Lonza) on gelatin-coated plates.

### Cell viability assay

H9-ECs were cultured in 96-well plate and cell viability analyses were performed using CCK8-based *in vitro* cell proliferation and cytotoxicity assay kit (Beyotime) according to the manufacturer’s instructions. Cells were incubated in the presence of 10 μl CCK8 reagent per well for 3 h. Absorbance at 450 nm was measured using a MD M5 SpectraMax reader (Molecular Devices).

### TUNEL assay

We detected apoptosis by TUNEL (Terminal deoxynucleotidyl transferase-mediated deoxyuridine triphosphase nick labeling) assay using an *In-situ* Cell Death Detection Kit (Roche) in accordance with the manufacturer’s instructions. H9-ECs were fixed with paraformaldehyde for 1 h, permeabilized with 0.1% Triton-X, and incubated with TUNEL reaction mixture for 1 h at 37 °C, and then incubated with nuclei fluorescent dye DAPI (Roche) for 5 min at room temperature. Images were collected and analyzed using Nikon TS100 series of inverted microscope.

### Caspase 3 activity assay

H9-ECs were cultured in 6-well plates. Caspase 3 activities were performed using Caspase 3 Assay Kit (Beyotime) according to the manufacturer’s instructions. Standard curve of pNA (p-nitroaniline) concentration relative to A405 was firstly generated. At least 1 × 10^7^ cells were collected by centrifugation at 600 g for 5 min at 4 °C. The cell pellets were washed with DPBS and re-suspended in 1× lysis buffer at a concentration of 100 μl per 2 × 10^7^ cells, incubated on ice for 15 min and then centrifuged at 16000–20000 g for 15 min at 4 °C. Concentration of proteins was measured by Bradford method. Appropriate amount of protein was put in a 96-well plate and 10 μl of Ac-DEVD-pNA (acetyl-Asp-Glu-Val-Asp p-nitroanilide) (2 mM) was added per well, and then incubated for 2 h at 37 °C. Absorbance at 405 nm was read using a MD M5 SpectraMax reader (Molecular Devices).

### Cell cycle and apoptosis assay

H9-ECs were cultured in 6-well plates and cell cycle and apoptosis was detected using a Cell Cycle and Apoptosis Analysis Kit (Beyotime) according to the manufacturer’s instructions. At least 1 × 10^5^ cells were collected by centrifugation at 1000 g for 5 min. Cell pellets were then washed with iced DPBS, re-suspended and fixed with 70% ethanol for 24 h. After centrifugation again, the cell pellets were re-suspended with 0.5 ml Propidium Iodide (PI) per tube and incubated in dark room for 30 min at 37 °C. The red fluorescence at 488 nm was detected by flow cytometry (Cytoflex). Images were collected and analyzed using Flowjo.7.6.1.Min.

### Tube formation assay

200 μl matrigel was plated in one well of 24-well plates and incubated for 30 min at 37 °C. 3 × 10^4^ H9-ECs were seeded and the formation of cord-like structures was assessed in 6 hours. 3–5 random fields in each well were imaged and counted under 10× phase contrast microscope and 3 independent experiments were performed in this study. The data were analyzed using Image J software. Specifically, we quantify the tube network by measuring the following two parameters: number of tubes, length of tubes. We choose a connection between two branch nodes as a tube, then mark all the tubes numerically, and the total number of tubes is counted. Angiogenesis Analyzer plugin16 in Image J software is utilized for quantification of tube network. Regarding analyzing the length of tubes, the graphs with marked number are opened by Image J and the length of tubes are measured manually using the length measurement function of Image J. The length of each tube is the distance between the two branch nodes, and the total length of tubes can be obtained by adding length of each tube together. Four representative images were used for the analyses in each experiment and three independent experiments were performed in this study.

### Cell proliferation assay

The cell proliferation assay was used to analyze the proliferation of endothelial cells following the manufacturer’s protocols (Cell Signaling Technology). The H9-ECs were incubated in EGM-2 medium along with BrdU solution for 6 hours at 37 °C. HRP conjugate substrate was subsequently added and the absorbance was read at 450 nm by MD M5 SpectraMax (Molecular Devices). All experiments were performed in triplicates and data were analyzed using GraphPad Prism 6.

### Wound healing assay

1 × 10^5^ H9-ECs were seeded in one well of 24-well plates overnight in EGM-2 medium with or without CdCl_2_. A linear scratch was generated by a sterile 200-μl plastic tip. Images were collected at 0 h and 12 h in each well and data were analyzed by Image J.

### Immunofluorescence staining

Cells were fixed with 4% paraformaldehyde for 15 min, permeabilized with 0.2% Triton X for 5 min, and blocked with 3% BSA for 1 hours. Cells were subsequently stained with appropriate primary antibodies (Abcam) and AlexaFluor conjugated secondary antibodies (Santa Cruz). Nuclei were stained with DAPI (Roche). The primary antibodies OCT4 (Santa Cruz), NANOG (Santa Cruz), SSEA-4 (Abcam) and SOX2 (Abcam) were used for pluripotency staining of undifferentiated H9 cells. The primary antibody of CD144 (Abcam) was used for staining of H9-ECs. H9-ECs were also stained with Dil-Ac-LDL (Thermo Fisher Scientific). Images were collected using an inverted confocal microscope (Nikon) and NIS-Elements AR software.

### Mitochondria/cytosol fractionation of H9-ECs

H9-ECs were cultured in T75 flasks, and 5 × 10^7^ cells were collected by centrifugation at 100–200 g for 5–10 min at room temperature. Mitochondria/cytosol fractionation was performed using a Mitochondria/Cytosol Fractionation Kit (Beyotime) according to the manufacturer’s instructions. Cells were re-suspended with 1 ml of 1× Cytosol Extraction Buffer Mix containing 1 mM PMSF, homogenized and then centrifuged at 600 g for 10 min at 4 °C. The supernatant was carefully collected which is a mixture of mitochondrial and cytoplasmic protein. Next, the supernatant was transferred to a new 1.5 ml tube, and centrifuged at 11,000 g for 10 min at 4 °C. The supernatant was collected and the pellet was saved. The supernatant was then transferred to a new 1.5 ml tube and centrifuged at 12,000 g for 10 min at 4 °C. The Supernatant is cytoplasmic protein. Mitochondrial protein lysate was obtained by re-suspending the saved pellet with 100 μl of the Mitochondrial Extraction Buffer Mix containing 1 mM PMSF.

### Western blot

H9-ECs were grown in 6-well plates to 80% confluence and detached with TrypLE (Gibco). Cells were pelleted at 12000 rpm for 3–5 min at 4 °C. After washing with DPBS, the pellets were re-suspended in 50–100 μl lysis buffer. Lysates were placed on ice for 30 min and then the supernatants were collected after centrifuging at 12000 rpm for 5 min. Protein concentration was measured by a BCA kit (Pierce). Western blot was performed using standard protocol with the following antibodies: Caspase 3 (Cell Signaling Technology, 1:1000), Caspase 9 (Beyotime, 1:1000), Bax (Cell Signaling Technology, 1:1000), Cytochrome c (Beyotime, 1:200), Bcl2 (Santa Cruz Biotechnology, 1:200), ERK1/2 (Cell Signaling Technology, 1:1000), P38 (Cell Signaling Technology, 1:1000), c-Myc (Cell Signaling Technology, 1:1000).

### RNA-Sequencing

RNA purity was checked using the Nano Photometer® spectrophotometer (IMPLEN), and RNA concentration was measured using Qubit^®^ RNA Assay Kit in Qubit^®^ 2.0 Flurometer (Life Technologies). RNA integrity was assessed using the RNA Nano 6000 Assay Kit of the Bioanalyzer 2100 system (Agilent Technologies). The transcriptome library for sequencing was generated using VAHTSTMmRNA-seq v2 Library Prep Kit for Illumina^®^ (Vazyme Biotech) following the manufacturer’s recommendations. The clustering of the index-coded samples was used VAHTS RNA Adapters set1/set2 for Illumina^®^ (Vazyme Biotech) according to the manufacturer’s instructions. After clustering, the libraries were sequenced on Illumina HiseqXTen platform using (2 × 150 bp) paired-end module. The raw images were transformed into raw reads by base calling using CASAVA (http://www.illumina.com/support/documentation.ilmn). Then, raw reads in a fastq format were first processed using in-house perl scripts. Clean reads were obtained by removing reads with adapters, reads in which unknown bases were more than 5% and low quality reads (the percentage of low quality bases was over 50% in a read, we defined the low quality base to be the base whose sequencing quality was no more than 10). At the same time, Q20, Q30, GC content of the clean data were calculated. After initial quality control, the clean reads were mapped to the reference sequence by using TopHat2 software (v2.1.1). The alignment files generated by TopHat2 were input to the Cufflinks software (v2.2.1), which is a program for the comparative assembly of transcripts and the estimation of their abundance in a transcriptome sequencing experiment by using the measurement unit FPKM (fragments per kilobase of transcript per million mapped reads). After using Cuffmerge program to merge transcripts of each sample in different materials and stages into a single gtf file that was used to identify differentially expressed genes, we used Cuffdiff program to find DEGs (differentially expressed genes). The differentially expressed genes were identified with q value ≤ 0.05 and a fold-change of ≥2 between two samples. Furthermore, cluster analysis, Gene Ontology (GO) enrichment analysis (GO::TermFinder), Pathway enrichment analysis (KOBAS) and Protein interaction analysis (based on StringDBdatabase) of differentially expressed genes were implemented if necessary.

### Compounds and solutions

Cadmium chloride (CdCl_2_) was purchased from Sigma-Aldrich, and stock solutions were prepared in 100 mM in H_2_O. When CdCl_2_ induction performed, a new vial of stock solution was used and dilutions were prepared within 30 min of induction. SB203580 (Selleck), PD0325901 (Selleck), SP600125 (Selleck), IWR-1 (Medchem Axon), and BIBX1382 (Sigma) were used in the signaling pathway analysis.

### Statistical analysis

Statistical significance was determined by unpaired two-tailed Student’s t-test to compare two groups and by One-way ANOVA to compare multiple groups. A *p* value of < 0.05 was considered statistical significant. Data were shown as mean ± sem and analyzed by GraphPad Prism 6 (GraphPad Software).

## Electronic supplementary material

Supplementary Information
