# Supplementary material for: Modeling cadmium-induced endothelial toxicity using human pluripotent stem cell-derived endothelial cells
Source: Sci Rep. 2017 Nov 1;7:14811. doi: 10.1038/s41598-017-13694-5 (PMC5665915; doi:10.1038/s41598-017-13694-5)
Supplement: Supplementary file 1 — Supplementary Information [file 41598_2017_13694_MOESM1_ESM.pdf]

Modeling cadmium-induced endothelial toxicity using human pluripotent stem cell-derived endothelial cells

Ling Tang[1, 2+], Jun su[1, 2+], Ping Liang[1, 2,*]

[1]Key Laboratory of combined Multi-organ Transplantation, Ministry of Public Health, the First Affiliated Hospital, Zhejiang University, 310003, Hangzhou, China

[2]Institute of Translational Medicine, Zhejiang University, 310029, Hangzhou, China

[*]**Correspondence:** Ping Liang, MD, PhD, 268 Kaixuan Road, North Block of the Central Building, Room 404, Hangzhou, China 310029. Phone: 86-571-86971872, E-mail: pingliang@zju.edu.cn

[+]These authors contributed equally to this work

SUPPLEMENTAL FIGURE LEGENDS

Supplemental Figure 1. Pluripotent staining of H9 hESCs using OCT4 (Green), SOX2 (Red), NANOG (Green) and SSEA4 (Red). DAPI indicates nuclear staining (Blue). Scale bar, 100 μm.

Supplemental Figure 2. Representative images of morphological changes in H9-ECs induced by 30 μM and 100 μM $CdCl_2$ for 24 h. Scale bar, 200 μm.

Supplemental Figure 3. Representative confocal images of TUNEL and DAPI staining in control and $CdCl_2$-treated (0.1-100 μM) H9-ECs. Scale bar, 200 μm.

Supplemental Figure 4. Full-length blots of Caspase 3 expression in control and $CdCl_2$-treated H9-ECs. Red boxes indicate the cropped blots shown in **Figure 3A**.

Supplemental Figure 5. Full-length blots of Caspase 9 expression in control and $CdCl_2$-treated H9-ECs. Red boxes indicate the cropped blots shown in **Figure 3B**.

Supplemental Figure 6. Full-length blots of Bax expression in control and $CdCl_2$-treated H9-ECs. Red boxes indicate the cropped blots shown in **Figure 3C**.

Supplemental Figure 7. Full-length blots of Bcl2 expression in control and $CdCl_2$-treated H9-ECs. Red boxes indicate the cropped blots shown in **Figure 3D**.

Supplemental Figure 8. Full-length blots of Bax-Mitochondria and Bax-cytosol expression in control and $CdCl_2$-treated H9-ECs. Red boxes indicate the cropped blots shown in **Figure 3E**.

Supplemental Figure 9. Full-length blots of Cytochrome c-cytosol and Cytochrome c-Mitochondria expression in control and $CdCl_2$-treated H9-ECs. Red boxes indicate the cropped blots shown in **Figure 3F**.

Supplemental Figure 10. Comparsion of cell cycle and apoptosis between control and $CdCl_2$-treated H9-ECs.

Supplemental Figure 11. Upper panel, representative images of tube formation on matrigel in control and 30 μM $CdCl_2$-treated H9-ECs assessed at 6 h. **Lower panel,** inverted images (black and white) to enhance the clarity of the representative figures in **Upper panel** using Adobe Photoshop. Scale bar, 200 μm.

Supplemental Figure 12. A and B. Upper panel, representative images of wound closure in control and 30 μM $CdCl_2$-treated H9-ECs assessed at 12 h. **Lower panel,** inverted images (black and white) to enhance the clarity of the representative figures in **Upper panel** using Adobe Photoshop. Scale bar, 200 μm.

Supplemental Figure 13. Bar graph to compare cell proliferation between control and $CdCl_2$-treated cells.

Supplemental Figure 14. A panel of most enriched GO analysis of three ontologies including biological process, cellular component, and molecular function.

Supplemental Figure 15. Representative confocal images showing the rescuing effect of $CdCl_2$-induced apoptosis in H9-ECs by a panel of inhibitors to block ERK, P38, JNK, Wnt and ErbB, respectively.

Supplemental Figure 16. Upper panel, representative confocal images showing the rescuing effect of $CdCl_2$-induced tube formation dysfunction phenotype in H9-ECs by a panel of inhibitors to block ERK, P38, Wnt and ErbB, respectively. **Lower panel,** inverted images (black and white) to enhance the clarity of the representative figures in **Upper panel** using Adobe Photoshop. Scale bar, 200 μm.

Supplemental Figure 17. A and B. Upper panel, representative confocal images showing the rescuing effect of $CdCl_2$-induced migration dysfunction phenotype in H9-ECs by a panel of inhibitors to block ERK, P38, Wnt and ErbB, respectively. **Lower panel,** inverted images (black and white) to enhance the clarity of the representative figures in **Upper panel** using Adobe Photoshop. Scale bar, 200 μm.

Supplemental Figure 18. Full-length blots of p-ERK expression in control and $CdCl_2$-treated H9-ECs. Red boxes indicate the cropped blots shown in **Figure 9A**.

Supplemental Figure 19. Full-length blots of p-P38 expression in control and $CdCl_2$-treated H9-ECs. Red boxes indicate the cropped blots shown in **Figure 9B**.

Supplemental Figure 20. Full-length blots of p-P38 expression in control H9-ECs, $CdCl_2$-treated H9-ECs, and H9-ECs treated with $CdCl_2$ and PD0325901 (ERK inhibitor). Red boxes indicate the cropped blots shown in **Figure 9C**.

Supplemental Figure 21. Full-length blots of c-Myc expression in control H9-ECs, $CdCl_2$-treated H9-ECs, and H9-ECs treated with $CdCl_2$ and SB203580 (P38 inhibitor). Red boxes indicate the cropped blots shown in **Figure 9D**.

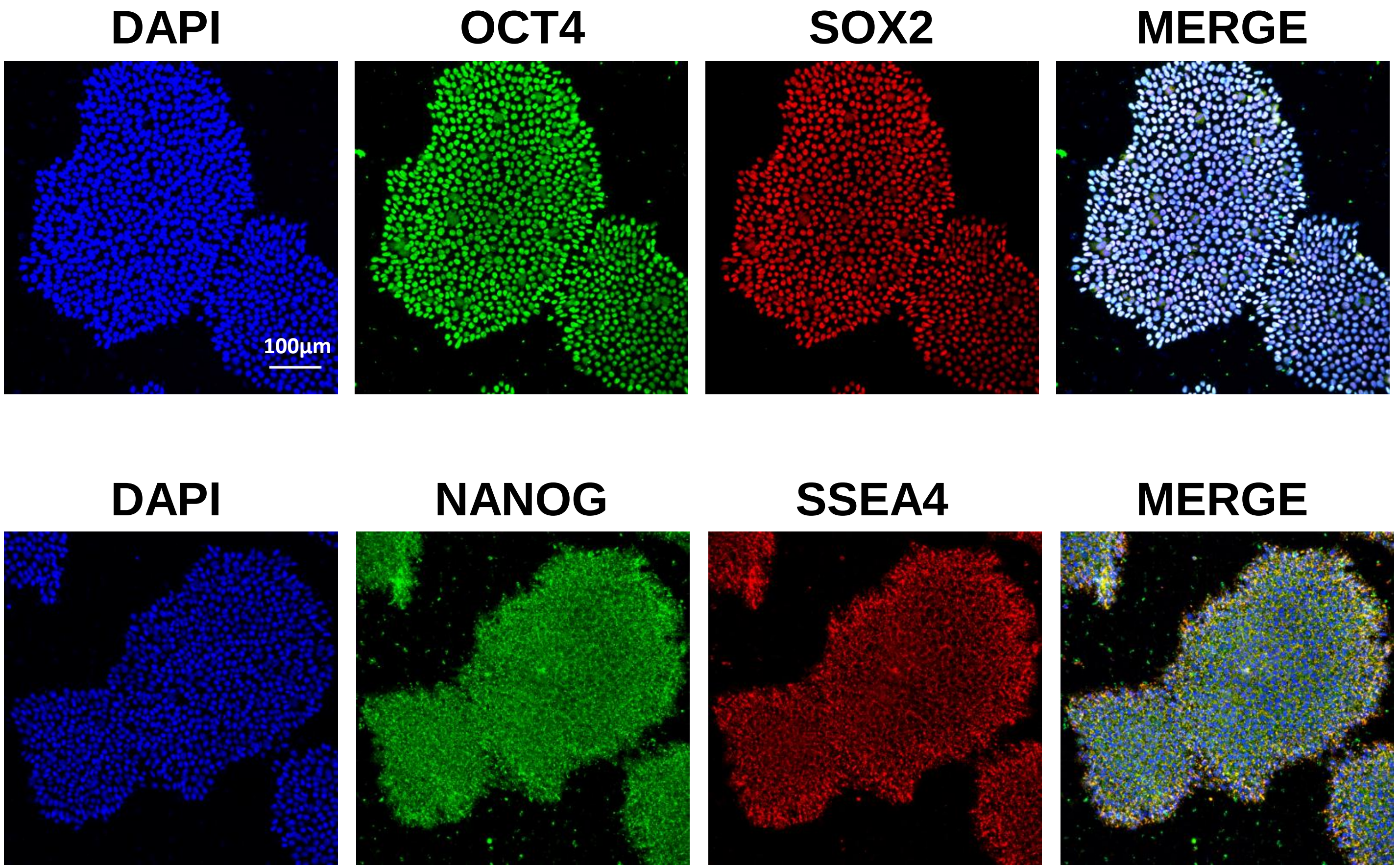

Supplemental Figure 1

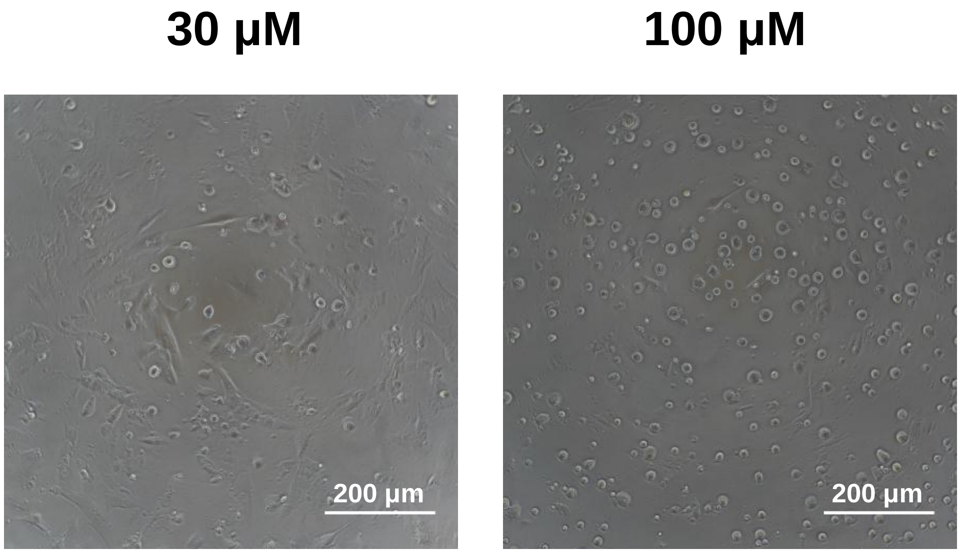

Supplemental Figure 2

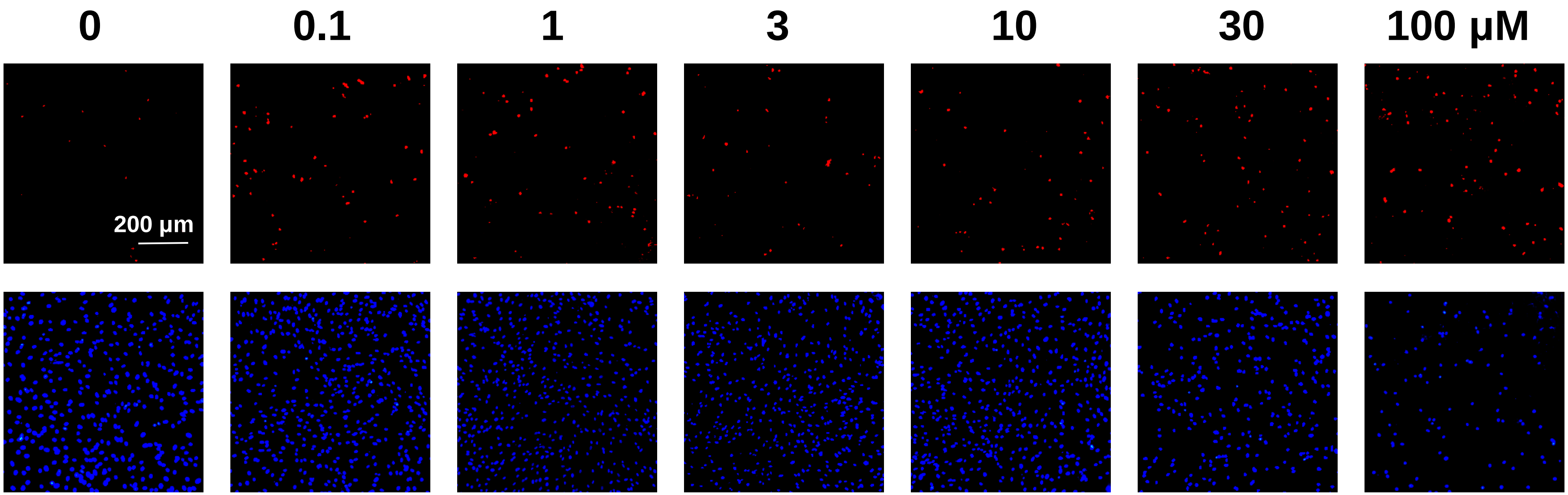

Supplemental Figure 3

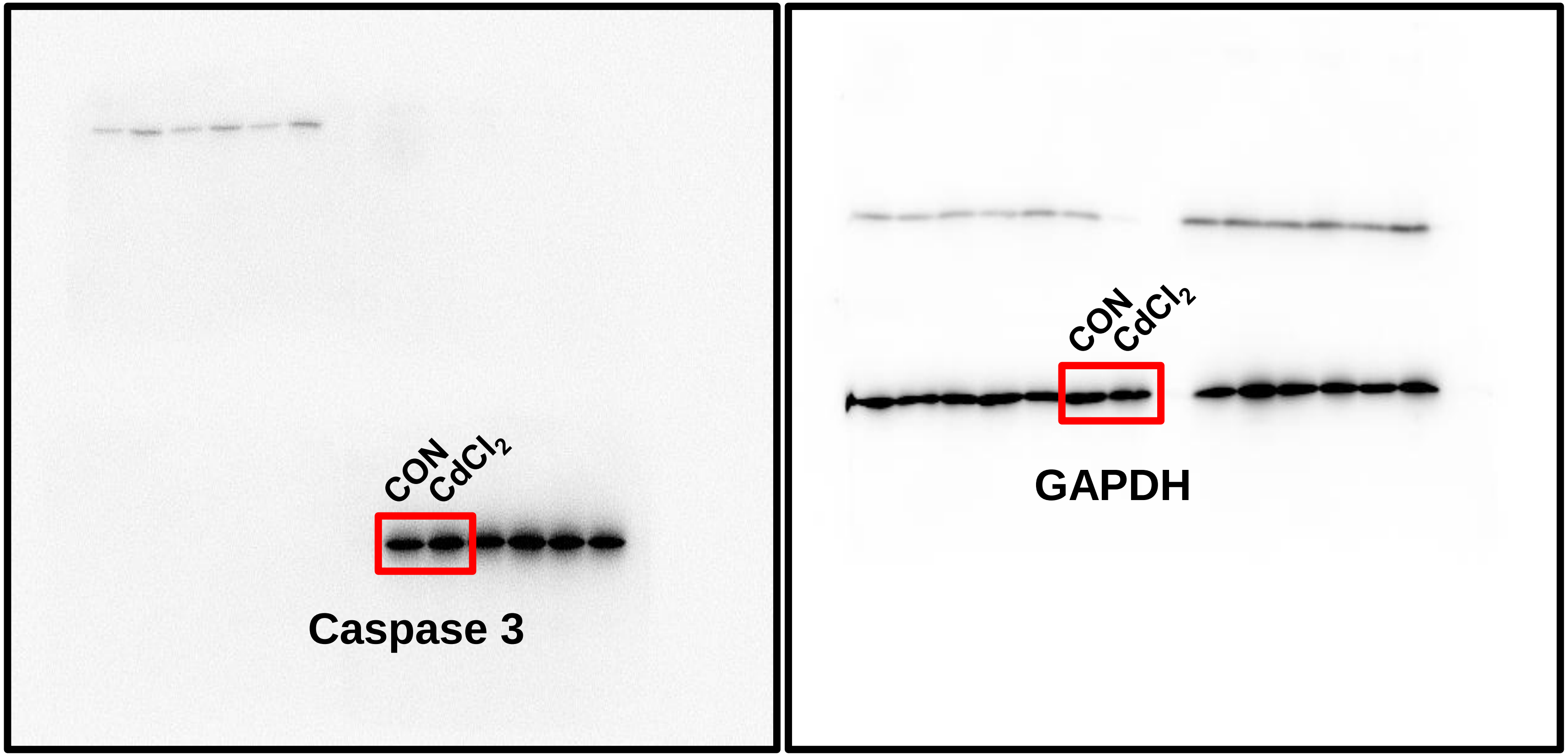

Supplemental Figure 4

B

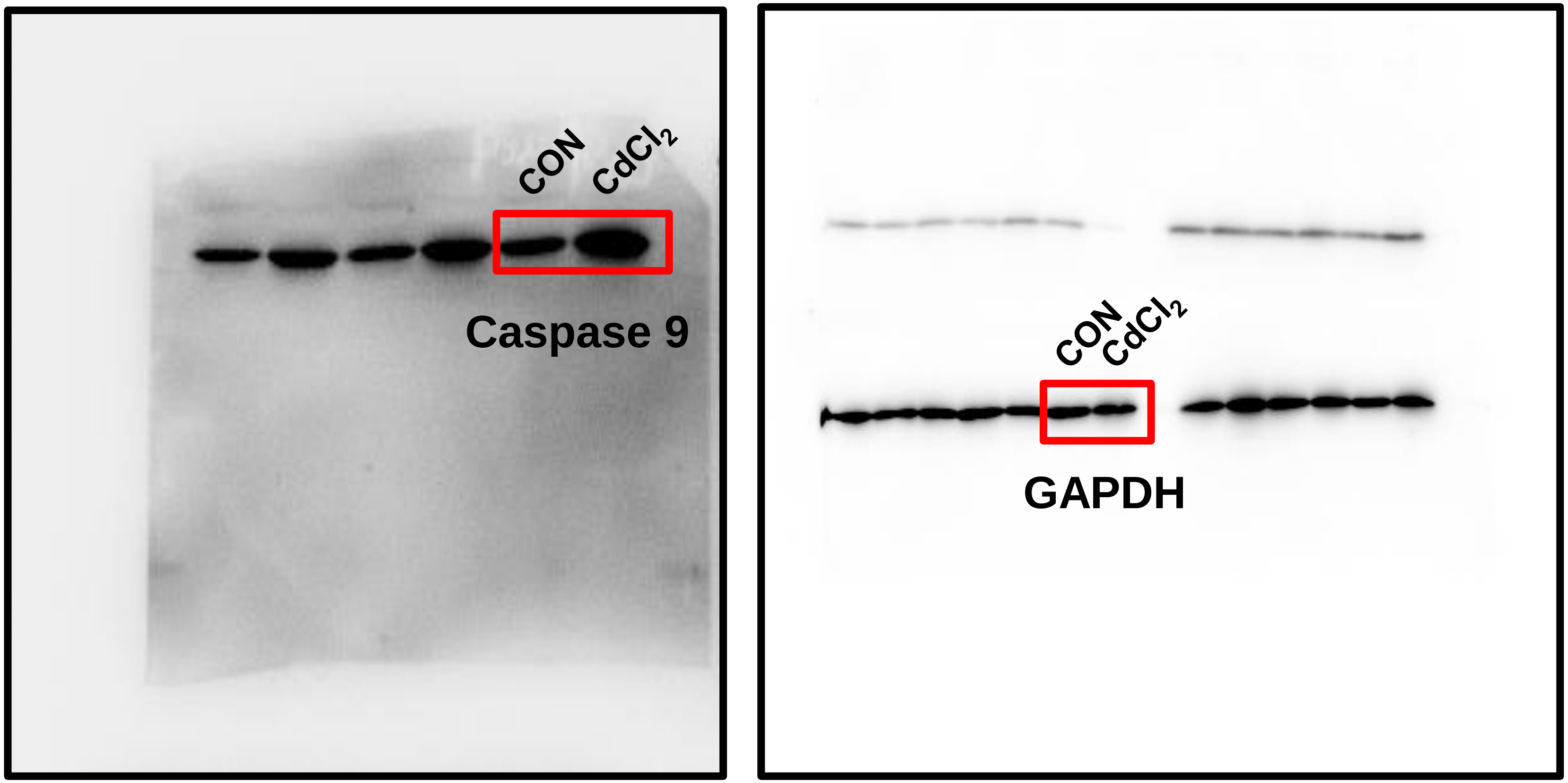

Supplemental Figure 5

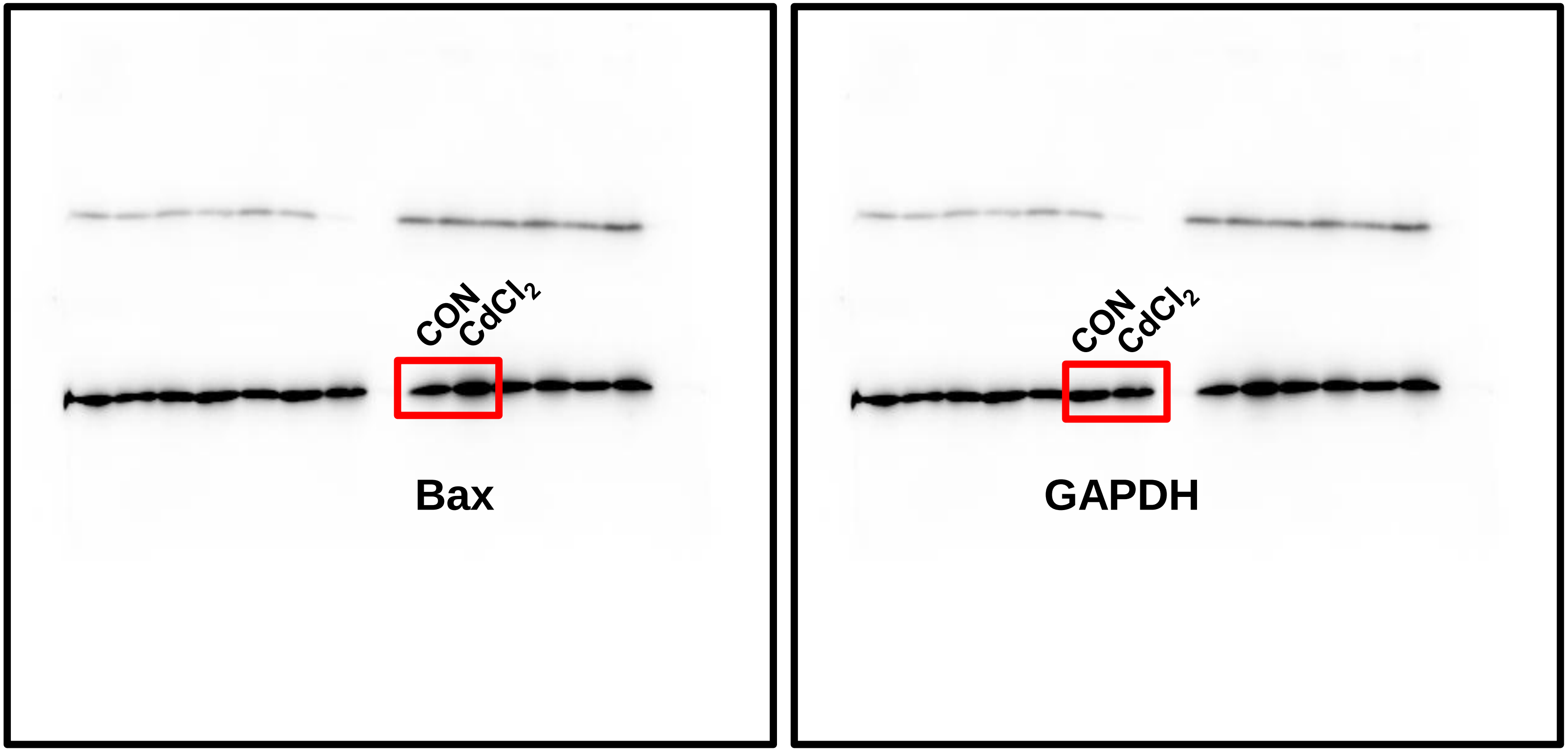

Supplemental Figure 6

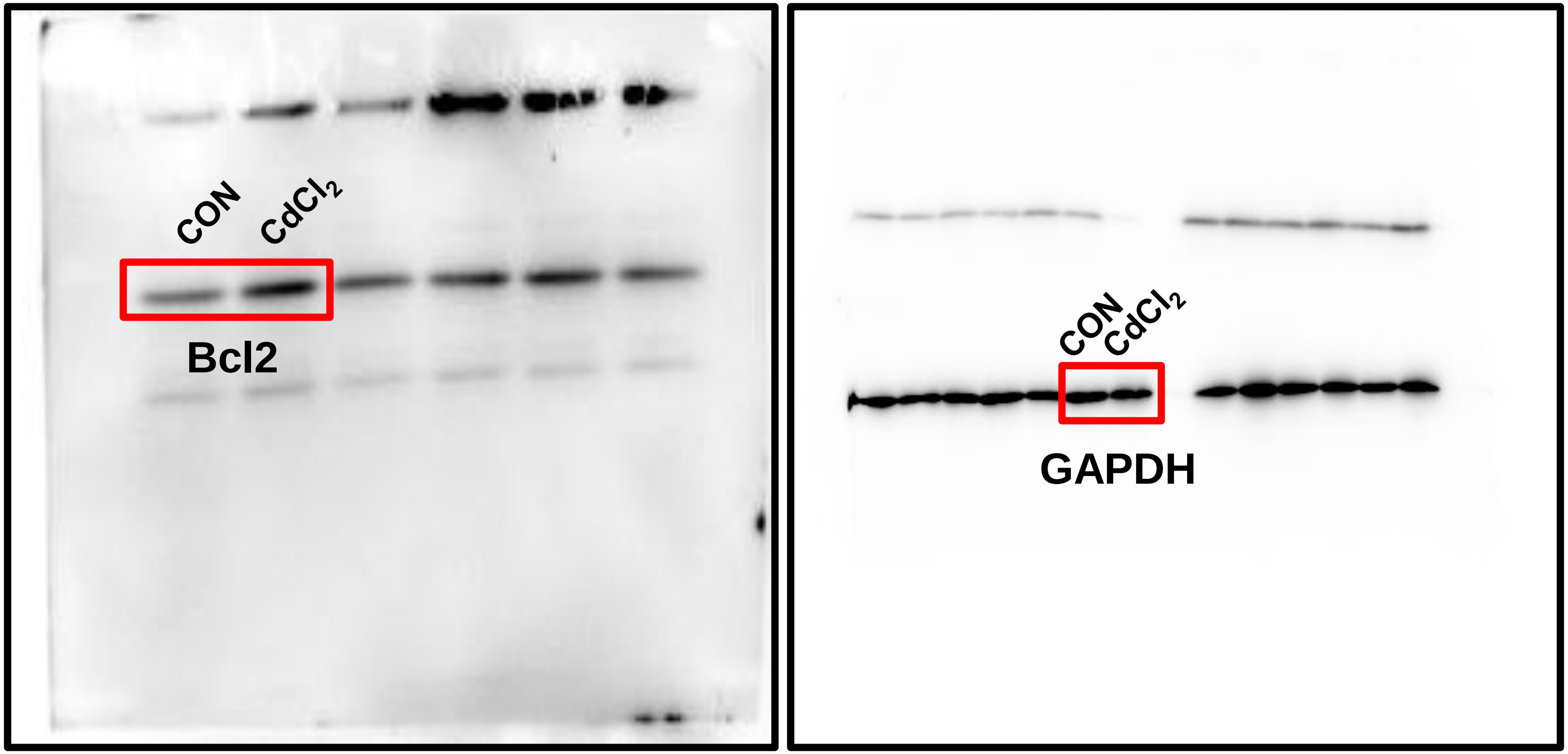

Supplemental Figure 7

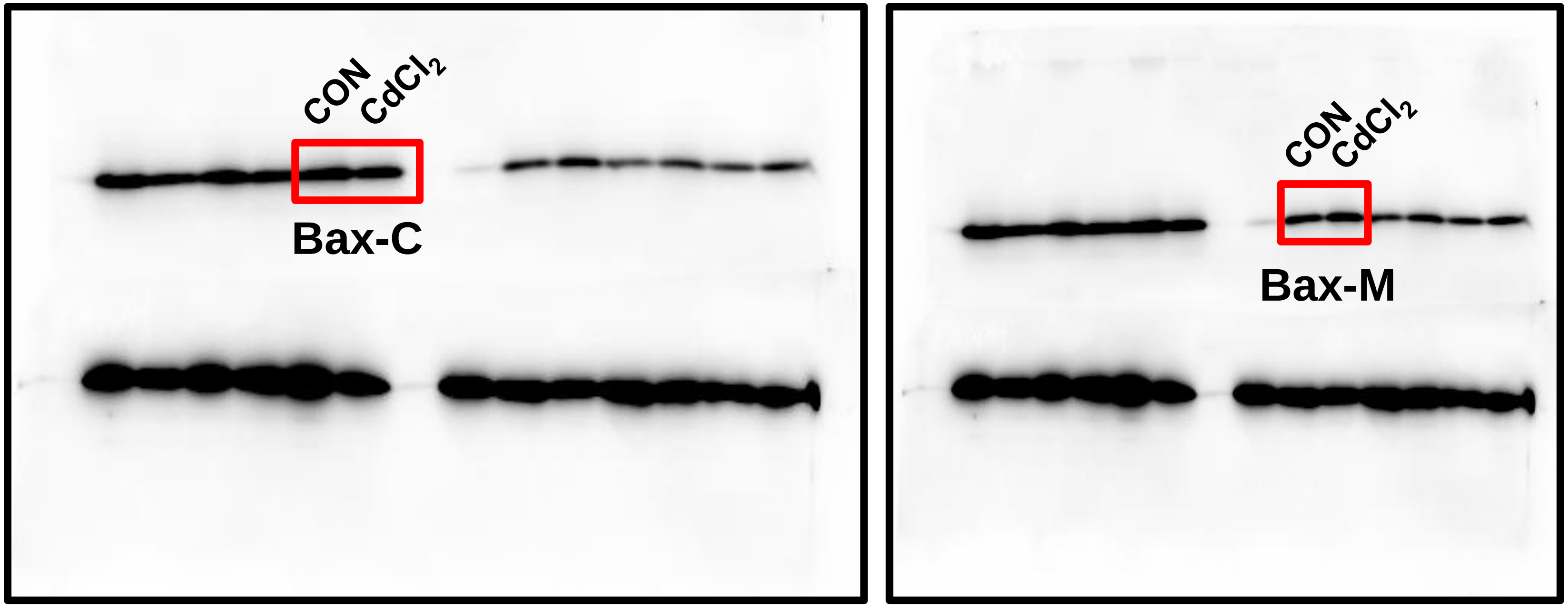

Supplemental Figure 8

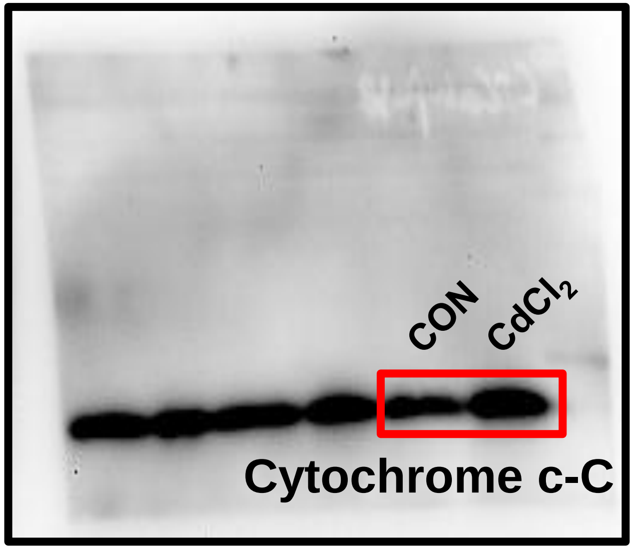

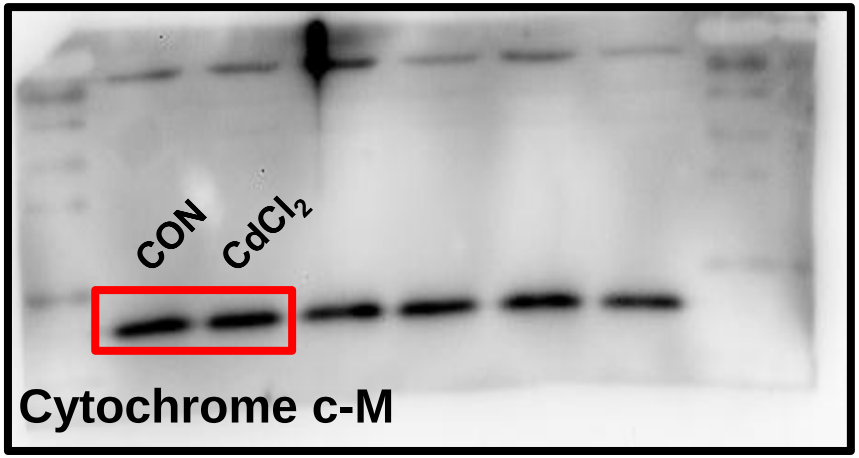

Supplemental Figure 9

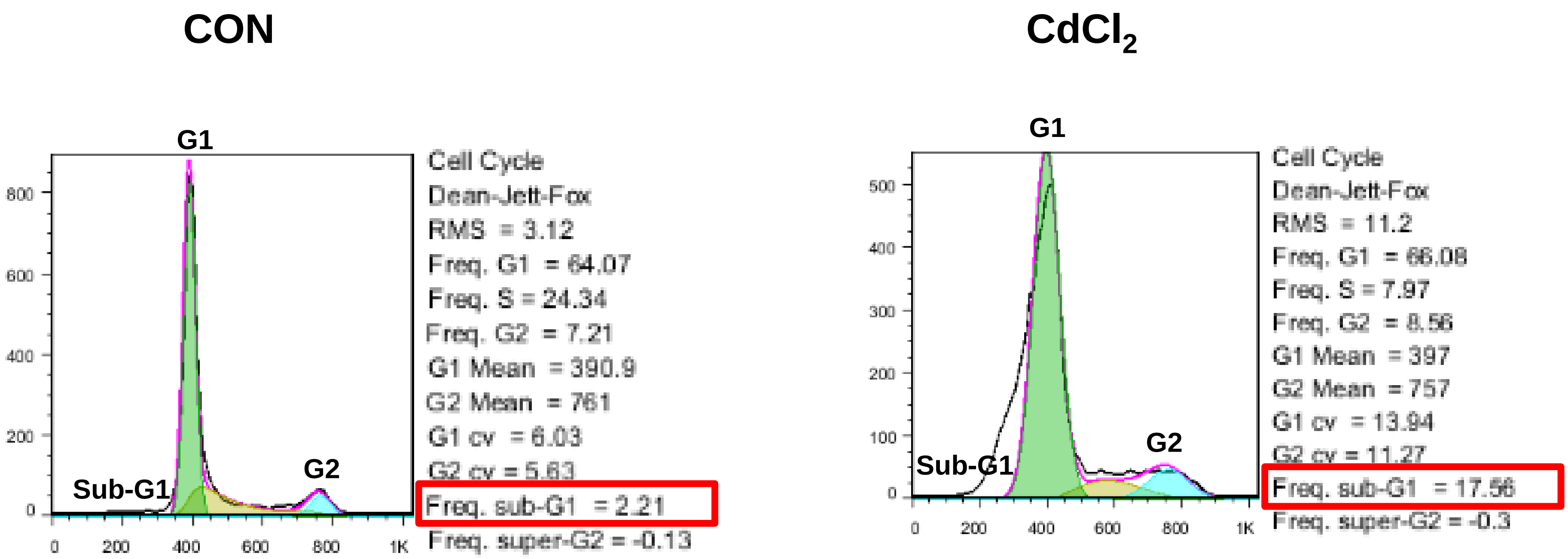

Supplemental Figure 10

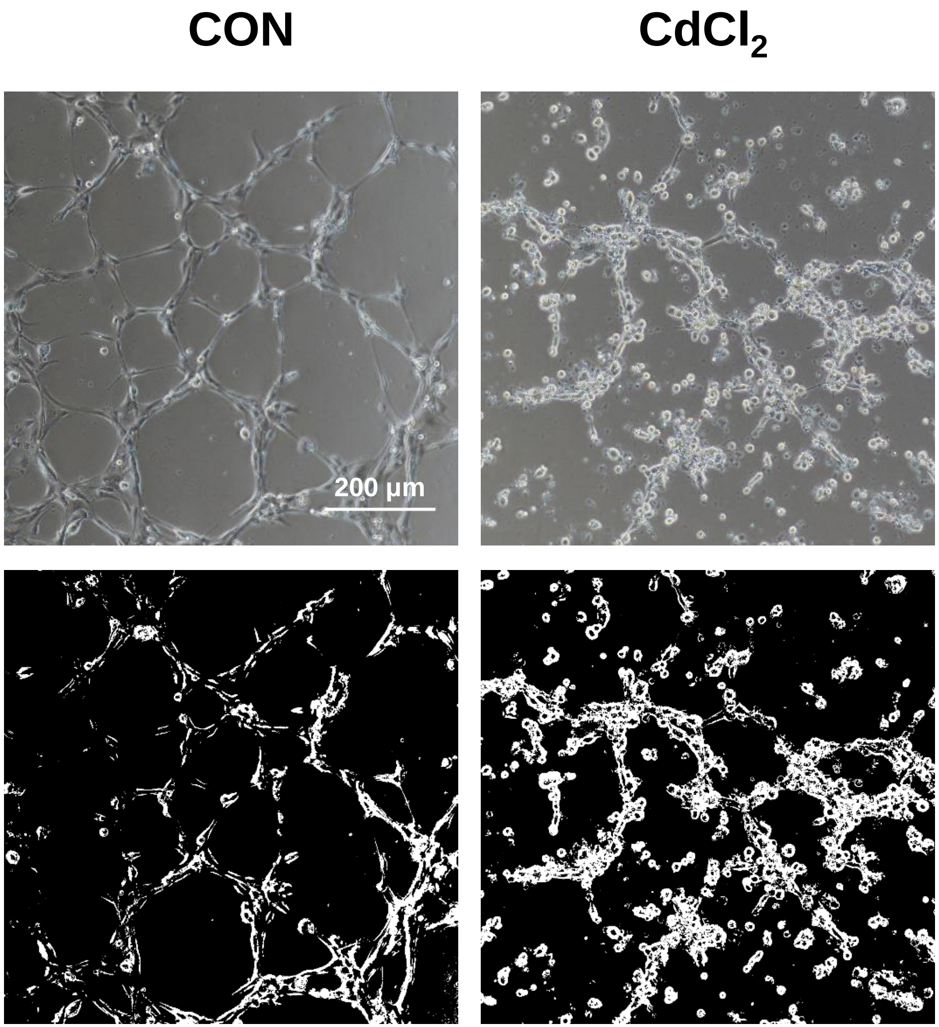

Supplemental Figure 11

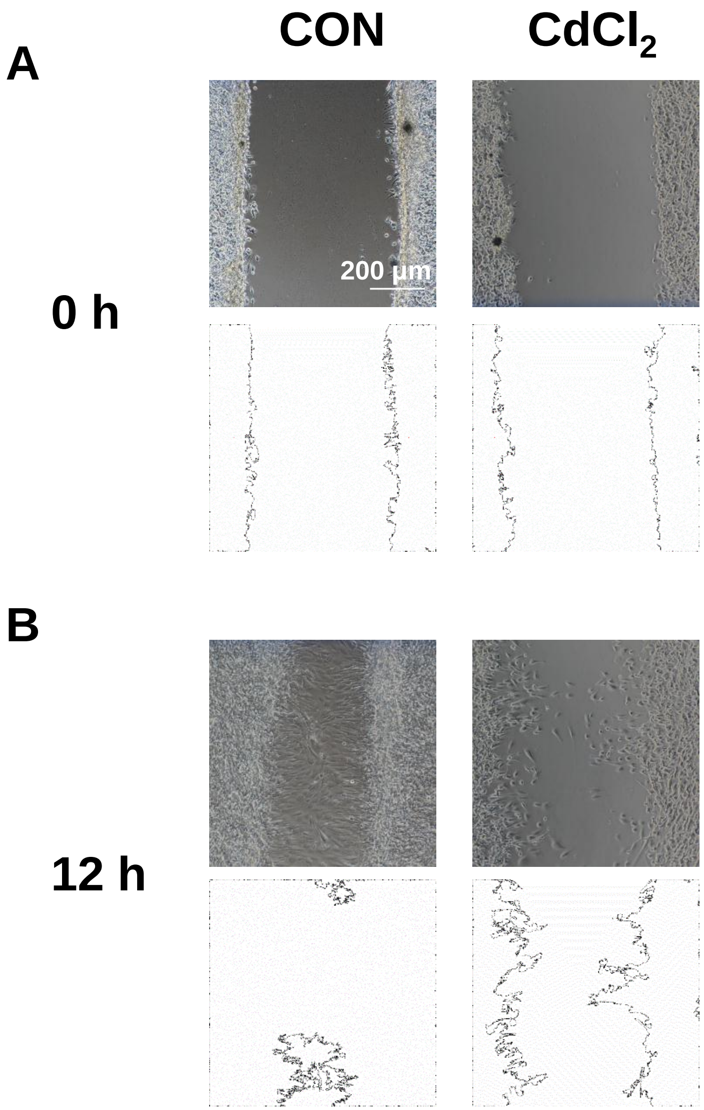

Supplemental Figure 12

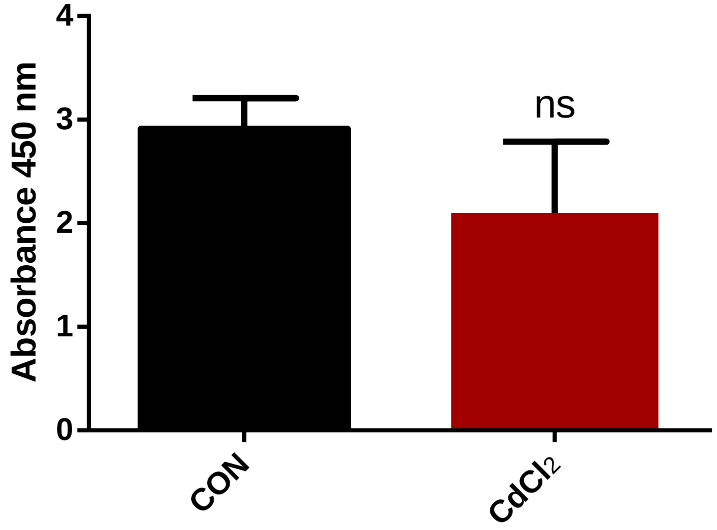

Supplemental Figure 13

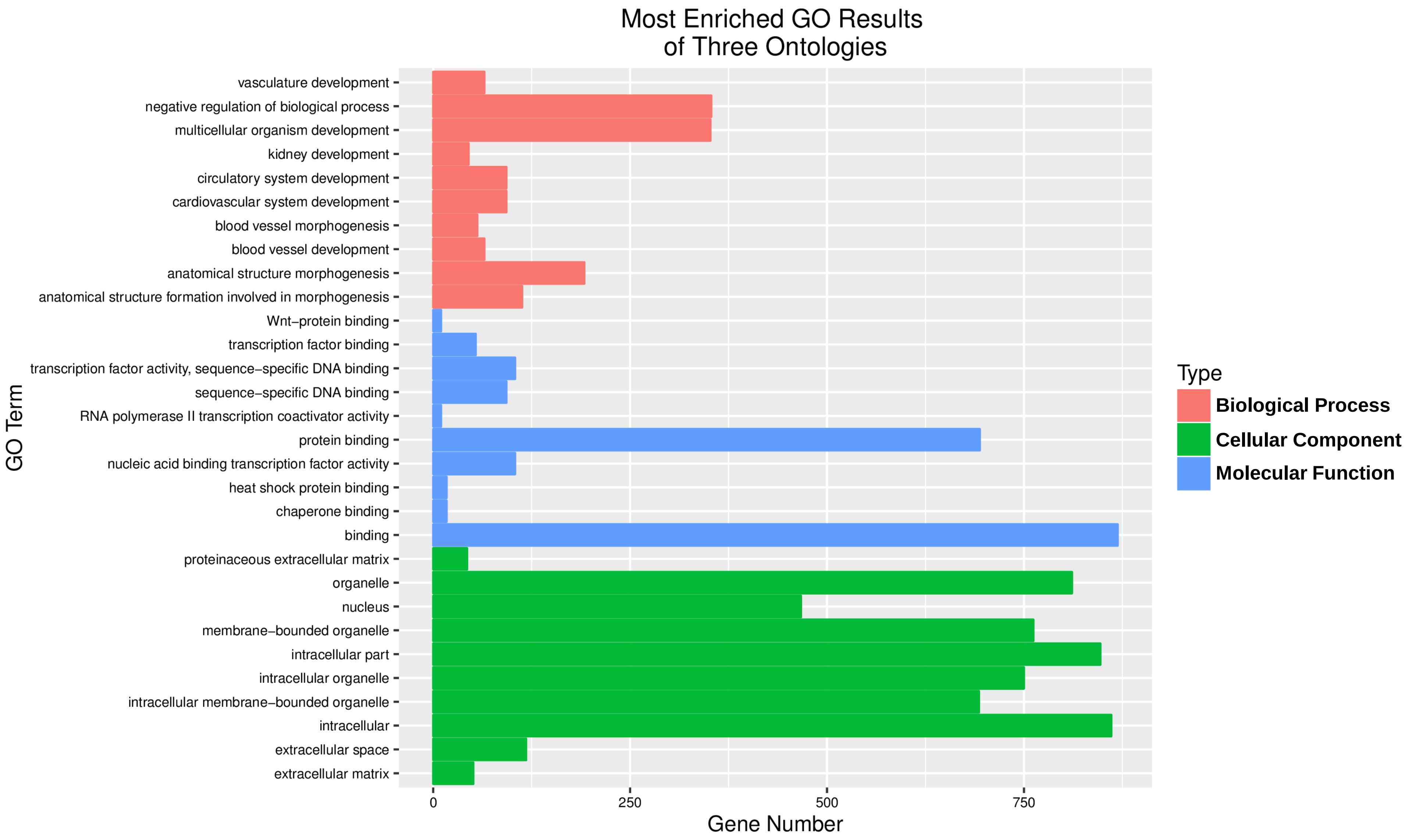

Supplemental Figure 14

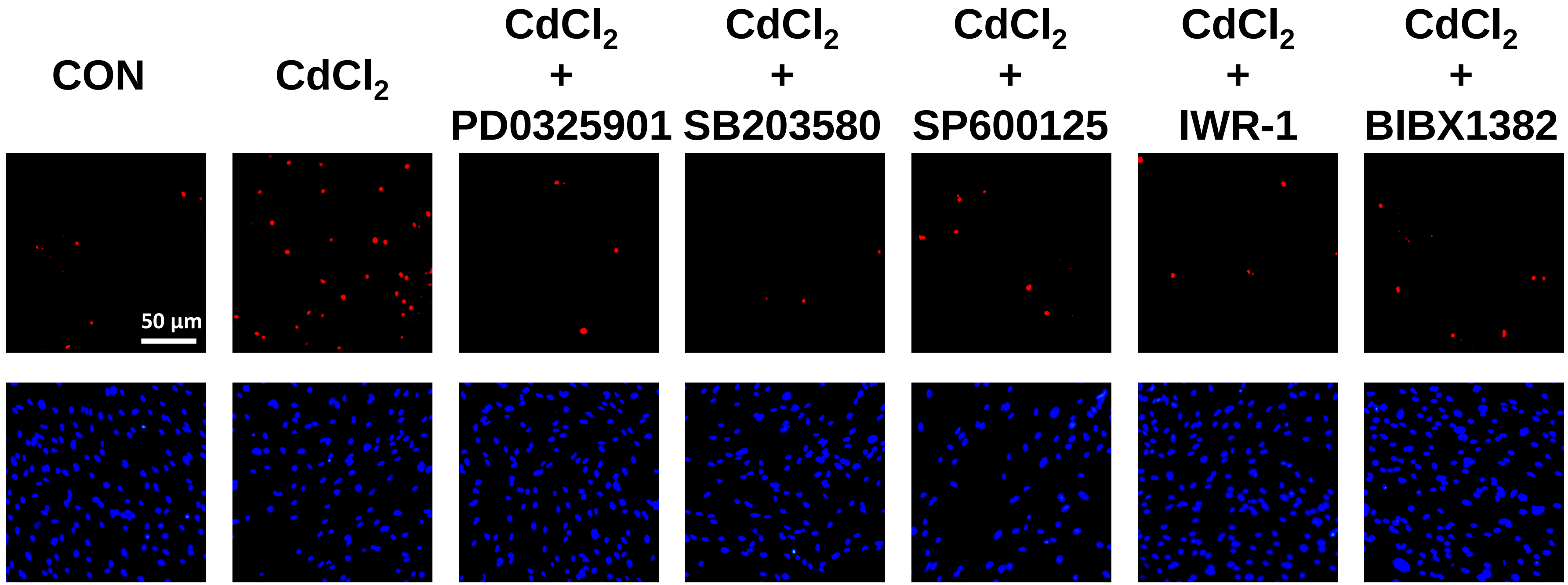

Supplemental Figure 15

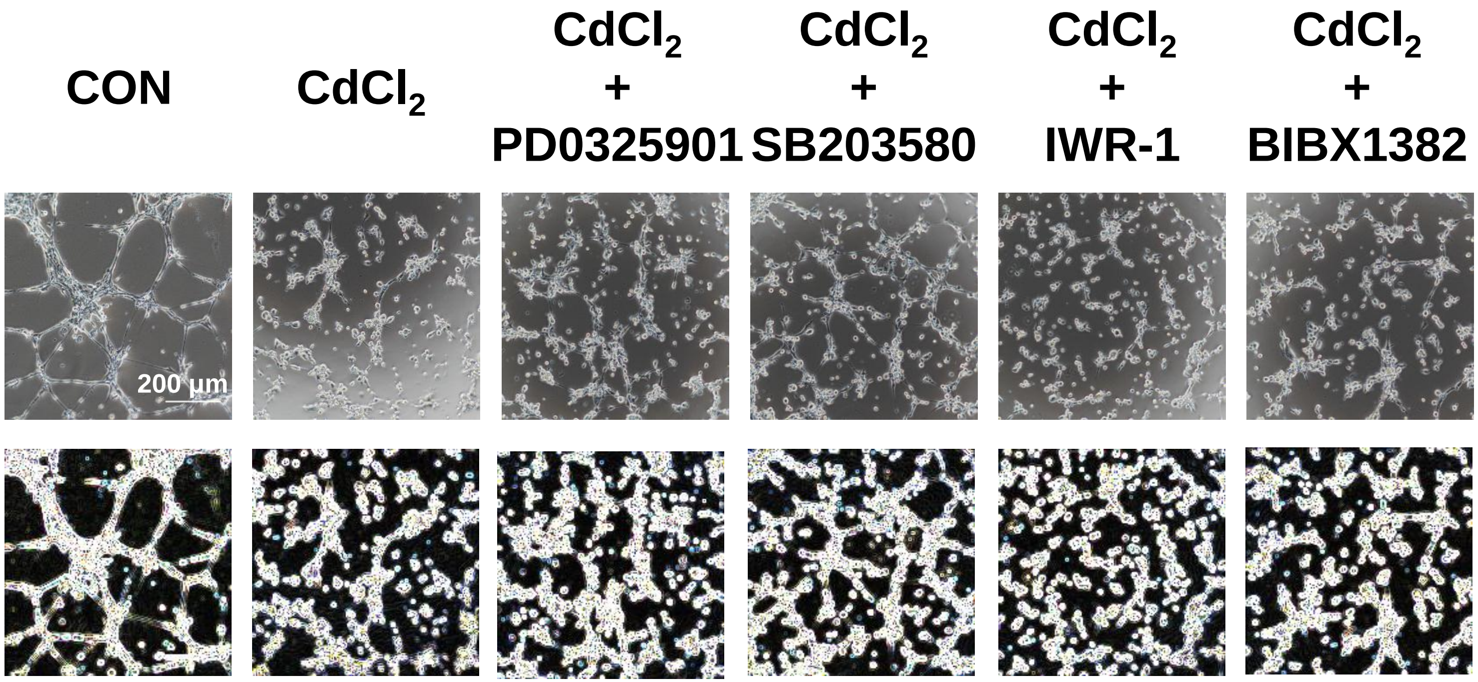

Supplemental Figure 16

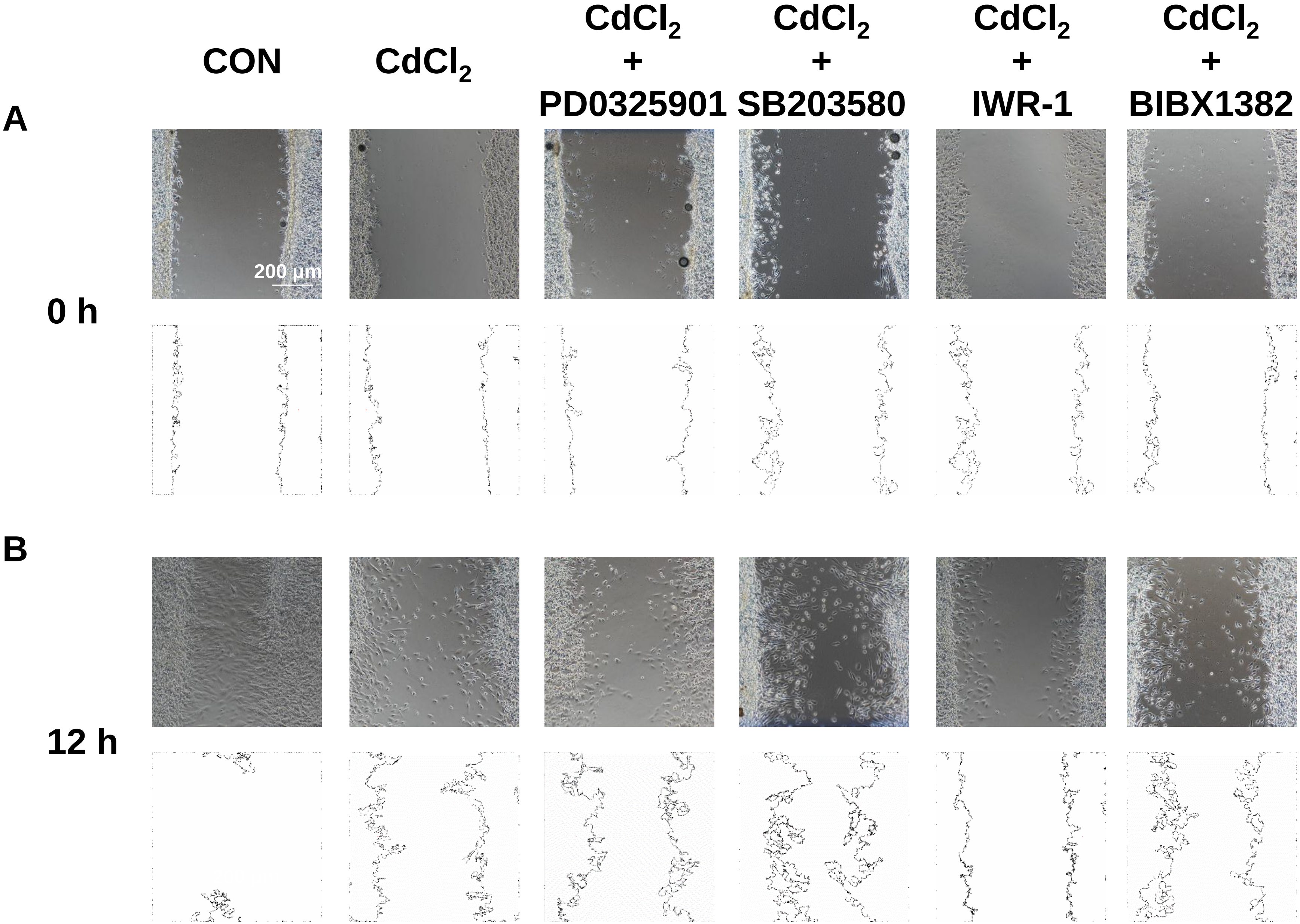

Supplemental Figure 17

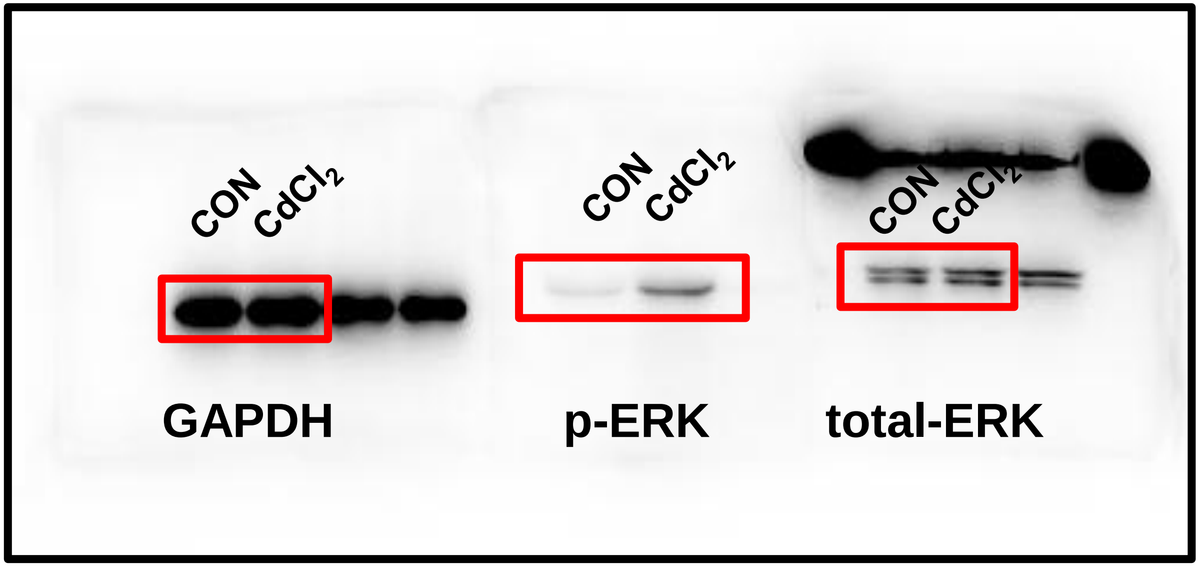

Supplemental Figure 18

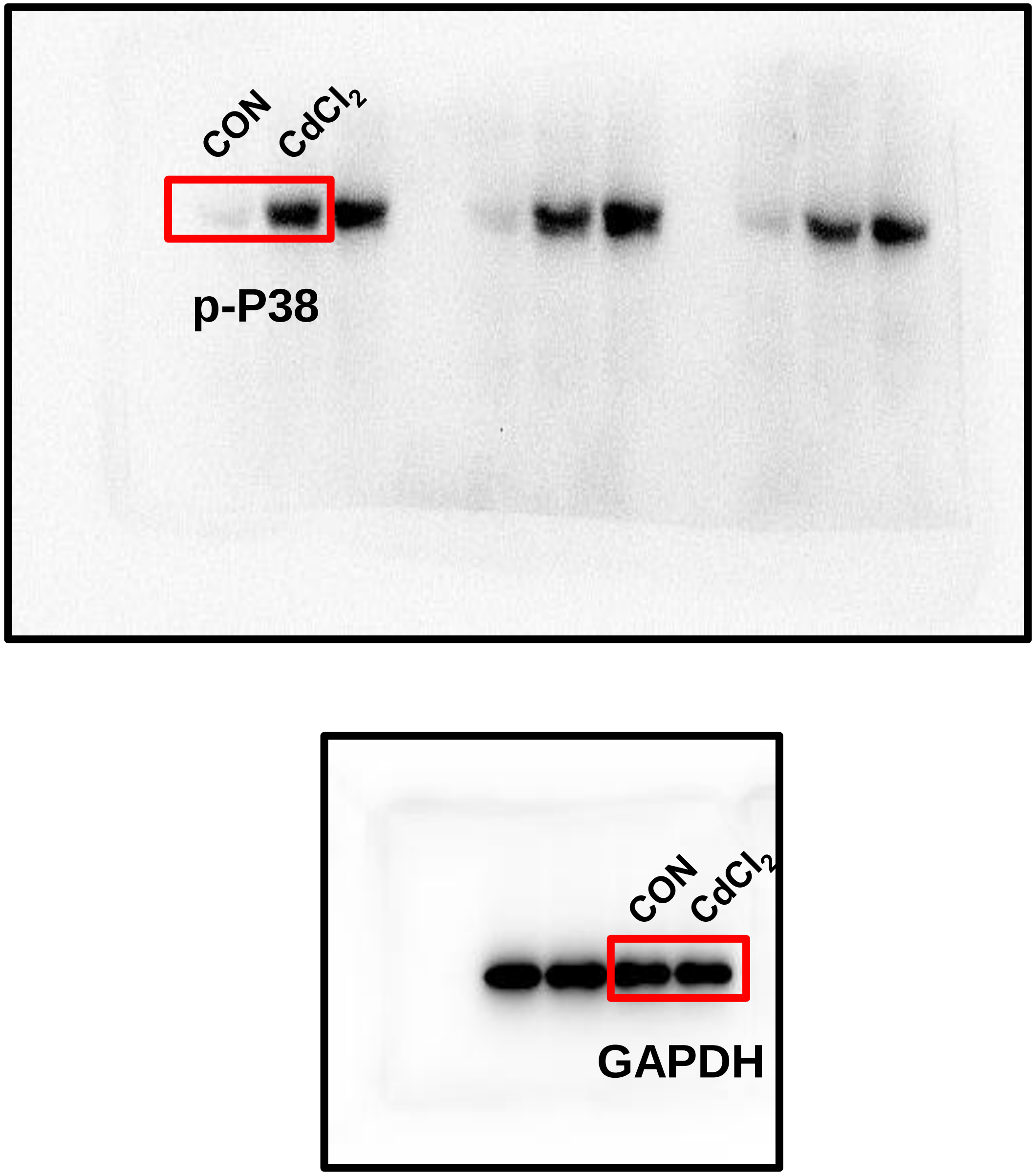

Supplemental Figure 19

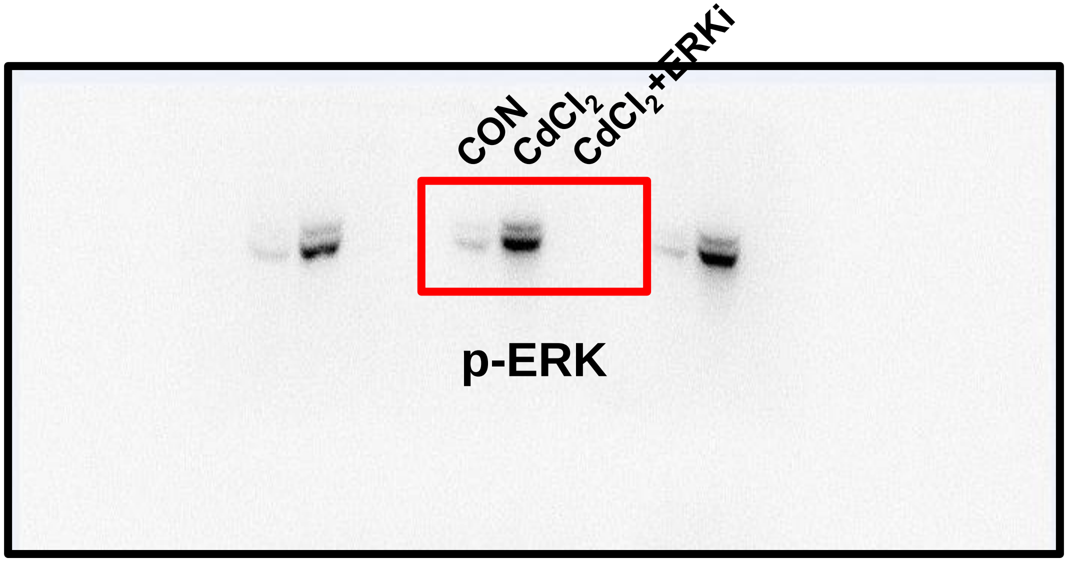

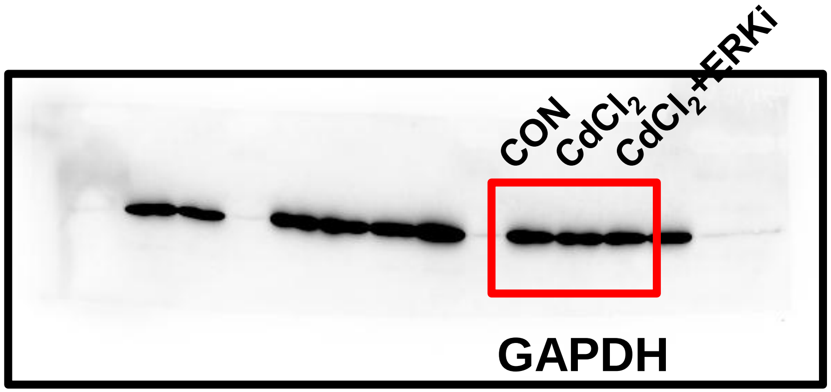

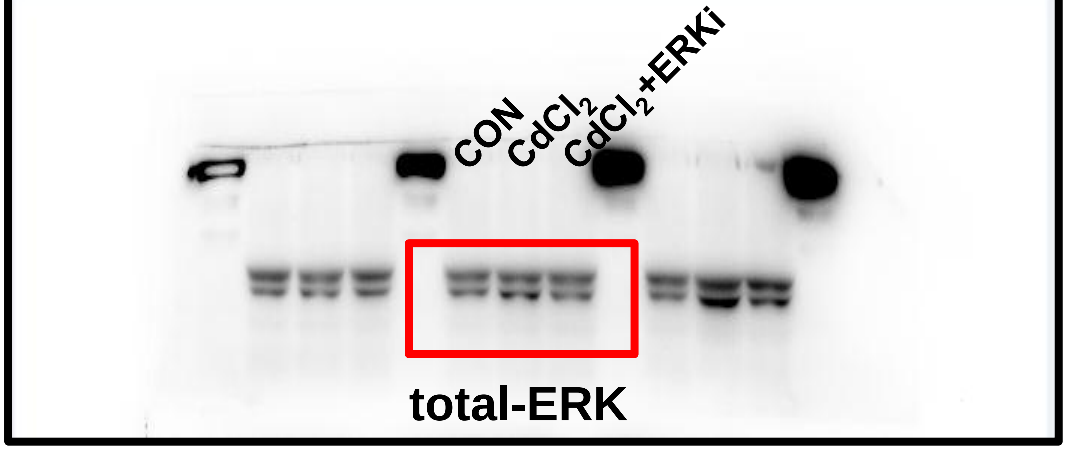

Supplemental Figure 20

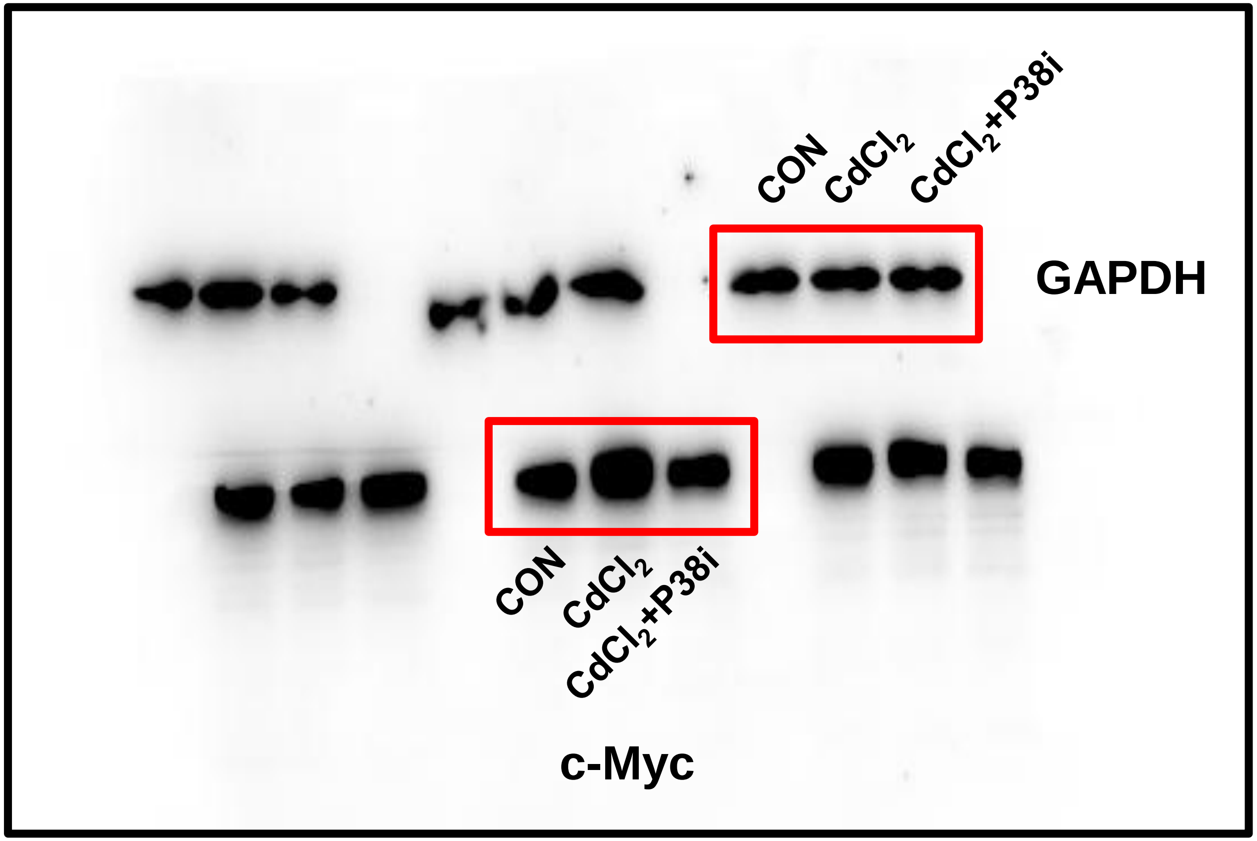

Supplemental Figure 21
